# Unlocking the Potential of Bi_2_S_3_‐Derived Bi Nanoplates: Enhanced Catalytic Activity and Selectivity in Electrochemical and Photoelectrochemical CO_2_ Reduction to Formate

**DOI:** 10.1002/advs.202400874

**Published:** 2024-05-17

**Authors:** Ahyeon Ma, Yongsoon Lee, Dongho Seo, Jiyoon Kim, Soohyeok Park, Jihoon Son, Woosuck Kwon, Dae‐Hyun Nam, Hyosung Lee, Yong‐Il Kim, Han‐Don Um, Hyeyoung Shin, Ki Min Nam

**Affiliations:** ^1^ Department of Chemistry and Institute for Future Earth Pusan National University Geumjeong‐gu Busan 46241 Republic of Korea; ^2^ Graduate School of Energy Science and Technology (GEST) Chungnam National University Daejeon 34134 Republic of Korea; ^3^ Department of Chemical Engineering Kangwon National University Chuncheon Gangwon‐do 24341 Republic of Korea; ^4^ Department of Energy Science and Engineering Daegu Gyeongbuk Institute of Science and Technology (DGIST) Daegu 42988 Republic of Korea; ^5^ Korea Research Institute of Standards and Science (KRISS) 267 Gajeong Yuseong Daejeon 34113 Republic of Korea; ^6^ Department of Measurement Engineering University of Science and Technology 217, Gajeong, Yuseong Daejeon 34113 Republic of Korea

**Keywords:** Bi nanoplates, CO_2_ reduction, formate production, photoelectrochemical reaction, silicon nanowires

## Abstract

Various electrocatalysts are extensively examined for their ability to selectively produce desired products by electrochemical CO_2_ reduction reaction (CO_2_RR). However, an efficient CO_2_RR electrocatalyst doesn't ensure an effective co‐catalyst on the semiconductor surface for photoelectrochemical CO_2_RR. Herein, Bi_2_S_3_ nanorods are synthesized and electrochemically reduced to Bi nanoplates that adhere to the substrates for application in the electrochemical and photoelectrochemical CO_2_RR. Compared with commercial‐Bi, the Bi_2_S_3_‐derived Bi (S‐Bi) nanoplates on carbon paper exhibit superior electrocatalytic activity and selectivity for formate (HCOO^−^) in the electrochemical CO_2_RR, achieving a Faradaic efficiency exceeding 93%, with minimal H_2_ production over a wide potential range. This highly selective S‐Bi catalyst is being employed on the Si photocathode to investigate the behavior of electrocatalysts during photoelectrochemical CO_2_RR. The strong adhesion of the S‐Bi nanoplates to the Si nanowire substrate and their unique catalytic properties afford exceptional activity and selectivity for HCOO^−^ under simulated solar irradiation. The selectivity observed in electrochemical CO_2_RR using the S‐Bi catalyst correlates with that seen in the photoelectrochemical CO_2_RR system. Combined pulsed potential methods and theoretical analyses reveal stabilization of the OCHO* intermediate on the S‐Bi catalyst under specific conditions, which is critical for developing efficient catalysts for CO_2_‐to‐HCOO^−^ conversion.

## Graphical abstract

The study demonstrates the transformation of Bi_2_S_3_ nanorods into Bi nanoplates (S‐Bi), yielding catalysts highly effective in CO_2_‐to‐HCOO^−^ conversion in electrochemical and photoelectrochemical systems. Defect states in the catalysts significantly aid HCOO^−^ production. S‐Bi/SiNWs photocathodes show remarkable activity and selectivity, suppressing H_2_ generation via pulsed electrolysis. These findings highlight the effectiveness of S‐Bi as a catalyst for CO_2_ reduction reactions.

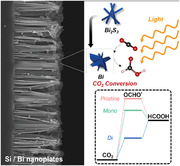

## 1 Introduction

The photoelectrochemical CO_2_ reduction reaction (PEC‐CO_2_RR) has recently attracted increasing attention for reducing carbon emissions and utilizing CO_2_ in various value‐added chemicals and fuels.^[^ ^1^, ^2^, ^3^, ^4^ ^]^ The PEC‐CO_2_RR typically occurs at the surface of the photocathode, whereas the oxygen evolution reaction occurs at the anode under solar‐light irradiation.^[^ ^5^ ^]^ To ensure high‐efficiency PEC‐CO_2_RR conversion, the ideal photocathode should be a p‐type semiconductor with a wide light‐absorption range, efficient carrier‐separation capabilities, and long‐term stability under light illumination.^[^ ^6^ ^]^ P‐type silicon (p‐Si) has great potential as a photocathode because of its narrow band gap (≈1.1 eV), and strong compatibility with established processes.^[^ ^7^ ^]^ Si nanostructures show good PEC‐CO_2_RR performance due to their high surface‐to‐volume ratio, which facilitates more efficient light absorption and catalyst loading.^[^ ^8^ ^]^ The resulting products include gaseous substances such as carbon monoxide, methane, and ethylene, as well as liquid compounds such as formate (HCOO^−^), methanol, and ethanol.^[^ ^9^, ^10^, ^11^, ^12^, ^13^, ^14^, ^15^ ^]^ The preference for liquid products over gaseous products in terms of storage and transportation convenience is well‐established.^[^ ^16^ ^]^ HCOO^−^ has emerged as a promising chemical‐based energy storage medium for hydrogen and as a key intermediate in renewable chemical feedstock applications.^[^ ^17^, ^18^ ^]^ However, current PEC‐CO_2_RR systems for HCOO^−^ synthesis are constrained by several limitations, including the intricate processes involved in forming multiple intermediates, the intense competition between the CO_2_RR and hydrogen evolution reaction (HER), and the lack of an adequate electrocatalyst for selective HCOO^−^ generation on the semiconductor surface.^[^ ^19^, ^20^ ^]^

In general, the electrochemical CO_2_ reduction process involves the formation of CO_2_ anion radical species that attach to the electrocatalyst, subsequently undergoing several steps depending on the catalyst type.^[^ ^21^, ^22^ ^]^ The control of product selectivity in electrochemical CO_2_ reduction (E‐CO_2_RR) has been extensively investigated.^[^ ^23^, ^24^, ^25^, ^26^ ^]^ Recently, electrocatalysts known for their selective production of CO and HCOO^−^ have been successfully utilized. The electrocatalyst on the photoelectrode, referred to as a co‐catalyst, plays a significant role in enhancing the separation of photo‐excited charges and regulating the catalytic selectivity of the PEC‐CO_2_RR system.^[^ ^27^ ^]^ However, there is no assurance that an efficient electrocatalyst for the E‐CO_2_RR will function equally effectively as a co‐catalyst on the semiconductor surface.^[^ ^28^ ^]^ Despite significant research efforts, the challenge remains in developing electrocatalysts that exhibit both selectivity and stability, while also being compatible with the Si surface for PEC‐CO_2_RR. Bi‐based materials are potential candidates for E‐CO_2_RR for HCOO^−^ generation due to their high selectivity for HCOO^−^.^[^ ^29^, ^30^, ^31^ ^]^ The morphological effects of Bi‐based catalysts have been investigated, aiming to enhance their activity and selectivity.^[^ ^32^, ^33^, ^34^ ^]^ However, the identification of the active sites in Bi‐based catalysts has received little attention. Specifically, examining the co‐catalytic properties of Bi on the Si surface and understanding the interaction between the Si and Bi in the PEC‐CO_2_RR system are crucial.

In this study, Bi_2_S_3_ nanorods are synthesized and transformed into Bi nanoplates for use in both the E‐CO_2_RR and PEC‐CO_2_RR. Electrochemically reduced Bi_2_S_3_ yields metallic Bi nanoplates with strong adhesion to conductive substrates. These Bi_2_S_3_‐derived Bi (S‐Bi) nanoplates on carbon paper show enhanced catalytic activity and selectivity for HCOO^−^ generation in the E‐CO_2_RR, achieving a Faradaic efficiency exceeding 93% and minimal H_2_ production over a wide potential range. Density functional theory calculations show that the distinctive defect states within the Bi catalyst, originating from S‐Bi, significantly contributed to stabilizing the OCHO* intermediate, thereby lowering the free‐energy barrier for the E‐CO_2_RR. Similarly, depositing these nanoplates on Si nanowires (SiNWs) produces a S‐Bi/SiNWs photocathode for the PEC‐CO_2_RR. Notably, the selectivity achieved with the S‐Bi catalyst in the E‐CO_2_RR is correlated with the performance of the PEC‐CO_2_RR system employing the S‐Bi/SiNWs photocathode. The robust adherence of the S‐Bi nanoplates to the Si substrate, coupled with their distinct catalytic properties, results in remarkable activity and selectivity for HCOO^−^ generation.

## 2 Results and Discussion

### 2.1 Synthesis and Characterization of Bi_2_S_3_ Nanorods and S‐Bi Nanoplates

**Figure 1:**
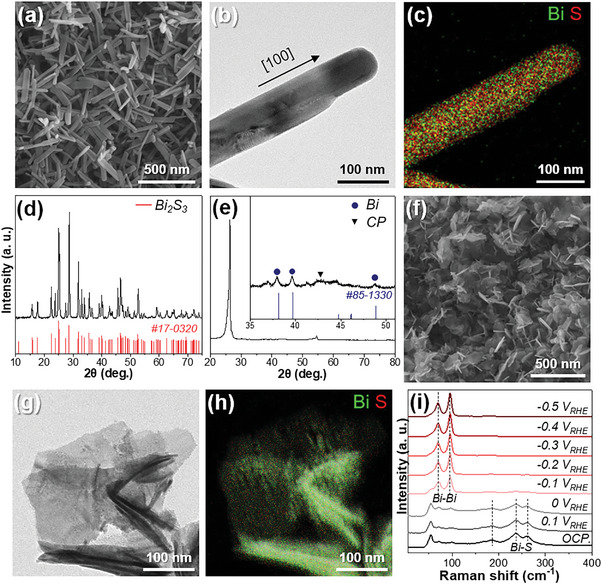
a) SEM and b) TEM image of Bi_2_S_3_ nanorods. c) TEM‐EDS elemental distributions of Bi_2_S_3_ and corresponding distributions of Bi (green) and S (red). XRD patterns of d) Bi_2_S_3_ and e) S‐Bi/CP. f) SEM and g) TEM images of S‐Bi nanoplates. h) TEM‐EDS elemental distributions of S‐Bi and corresponding distributions of Bi (green) and S (red). i) In situ Raman spectra of Bi_2_S_3_/CP as a function of the applied potentials in the CO_2_‐saturated 0.1 m KHCO_3_ electrolyte.

Bi_2_S_3_ nanorods were prepared by a facile hydrothermal method, with Bi_2_O_3_ and thiourea (SC(NH_2_)_2_) serving as the Bi and S sources, respectively. Due to the extremely small solubility product of Bi_2_S_3_ (*K_sp_* = 1.0 × 10^−97^),^[^ ^35^ ^]^ Bi_2_O_3_ undergoes sulfidation during the hydrothermal process. This process involves the etching of Bi_2_O_3_ to Bi^3+^ and subsequent crystallization with S^2−^,^[^ ^36^ ^]^ leading to the rapid growth of Bi_2_S_3_ nanorods. Scanning electron microscopy (SEM) image showed Bi_2_S_3_ nanorods with a length of 249 ± 26 nm and a width of 42 ± 9 nm (**Figure** **1**a). The distribution of Bi and S was evaluated by transmission electron microscopy (TEM) energy‐dispersive X‐ray spectroscopy (EDS) elemental mapping of a single nanorod (Figure 1b,c). The elemental map shows a homogeneous distribution of Bi and sulfur (S) atoms throughout the nanorods. EDS analysis indicated an atomic ratio representing the 2:3 conformation of Bi_2_S_3_. X‐ray diffraction (XRD) was used to determine the crystal structure of the Bi_2_S_3_ nanorods (Figure 1d). The diffraction patterns matched well with those of orthorhombic Bi_2_S_3_ (ICDD file no. 17–0320). The high‐resolution TEM (HRTEM) image of Bi_2_S_3_ shows the lattice spacing of the (220) plane of the orthorhombic phase (Figure S1, Supporting Information). The corresponding Fast‐Fourier transform (FFT) data showed that the nanorods were single‐crystalline in nature. The oxidation state of Bi_2_S_3_ was investigated using X‐ray photoelectron spectroscopy (XPS) (Figure S2, Supporting Information). The peaks at 158.7 and 164.0 eV were assigned to the Bi 4f_7/2_ and Bi 4f_5/2_ states, and the Bi 4f spectrum exhibited spin‐orbit components separated by 5.3 eV, indicating the presence of Bi^3+^.^[^ ^37^ ^]^ The peaks at 161.1 and 162.4 eV were assigned to the S 2p_3/2_ and S 2p_1/2_ states, respectively, suggesting the presence of S^2−^, and were consistent with the Bi_2_S_3_ composition. The electrochemical phase transition of Bi_2_S_3_ on the electrode surface (either carbon paper (CP) or F‐doped tin oxide (FTO)), was investigated through an electrochemical reduction process in 0.1 m KHCO_3_ solution. The Bi_2_S_3_ phase was transformed to metallic Bi by applying a potential of −2.0 V versus RHE for 1 h in 0.1 m KHCO_3_. With the application of negative potential, S^2−^ was rapidly released, accompanied by the reduction of Bi_2_S_3_ to Bi^0^, with structural rearrangement: Bi_2_S_3_ + 6e^−^ → 2Bi + 3S^2−^. Figure 1e shows the XRD patterns of the transformed metallic Bi, which matched well with the hexagonal phase of Bi (ICDD file no. 85–1330). The SEM image showed a nanoplate‐like morphology with side lengths of 138 ± 21 nm (Figure 1f). The reduction of Bi_2_S_3_ to metallic Bi initiates a compositional change, which affects material stability and induces a morphological transition. Surface energy plays a crucial role in determining the preferential growth of specific morphologies,^[^ ^38^ ^]^ ultimately influencing the observed transition from nanorods to alternative shapes such as nanoplates. The TEM image of the Bi nanoplate indicated a narrow, 2D structure (Figure 1g; Figure S3, Supporting Information). The structures of the Bi nanoplates contain numerous sub‐nanopores. The HRTEM images of the Bi nanoplate indicated a lattice spacing of 0.24 nm, which matched well with the (104) plane of hexagonal Bi (Figure S4, Supporting Information). The XPS data also indicated the presence of metallic Bi in the nanoplates (Figure S5, Supporting Information). The XRD results indicated a complete reduction to Bi; however, residual S was observed in the TEM‐EDS analysis (Figure 1h). A small amount of S at a concentration of at least 0.3 wt.% was consistently detected, even in the long‐term reduction reaction, in which the residual S is consistent with prior reports.^[^ ^39^, ^40^, ^41^ ^]^ Additionally, SEM‐EDS confirmed consistent elemental distribution, with the S content remaining at ≈0.4 wt.% (Figure S6a, Supporting Information). Subsequently, inductively coupled plasma optical emission spectroscopy (ICP‐OES) analysis was conducted to precisely quantify the amount of S atoms, revealing a concentration of ≈0.66 wt.% (Figure S6b, Supporting Information). According to the analysis methods, the Bi nanoplate exhibited uniform doping with S atoms, with a concentration ranging from ≈0.3 to 0.7 wt.%. This suggests that the Bi sites retain a partial non‐zero valence state during electrochemical reduction.^[^ ^42^ ^]^ The S‐doped Bi nanoplates on CP are simply denoted as S‐Bi/CP. However, the directly synthesized Bi nanoparticles depicted in Figure S7 (Supporting Information) showed no detectable presence of S atoms in either TEM‐EDS or SEM‐EDS analysis.

To observe structural reconstruction without interference from the air, in situ Raman spectroscopy analysis was employed to monitor the real‐time transformation of Bi_2_S_3_ to Bi under electrochemical reduction conditions in CO_2_‐saturated 0.1 m KHCO_3_, as depicted in Figure 1i. Raman peaks at 186, 238, and 262 cm^−1^, corresponding to the stretching modes of the Bi─S bond in Bi_2_S_3_, were observed at the open circuit potential (OCP).^[^ ^43^ ^]^ Upon applying a negative potential above −0.1 V versus RHE, these three peaks immediately diminished, while two new Raman peaks emerged at 72 and 97 cm^−1^, attributed to Bi–Bi vibrations.^[^ ^40^, ^44^ ^]^ Hence, in situ Raman spectroscopy provides direct evidence that Bi_2_S_3_ readily converts to metallic Bi during the electrochemical reduction process. This in situ observation is consistent with the phase transformation to metallic Bi as evidenced by XRD and HRTEM results. Unfortunately, we were unable to detect the interaction between Bi and CO_2_ leading to formate generation during CO_2_ reduction.

### 2.2 Electrochemical CO_2_ Reduction Reaction (E‐CO_2_RR) of Bi Electrodes

**Figure 2:**
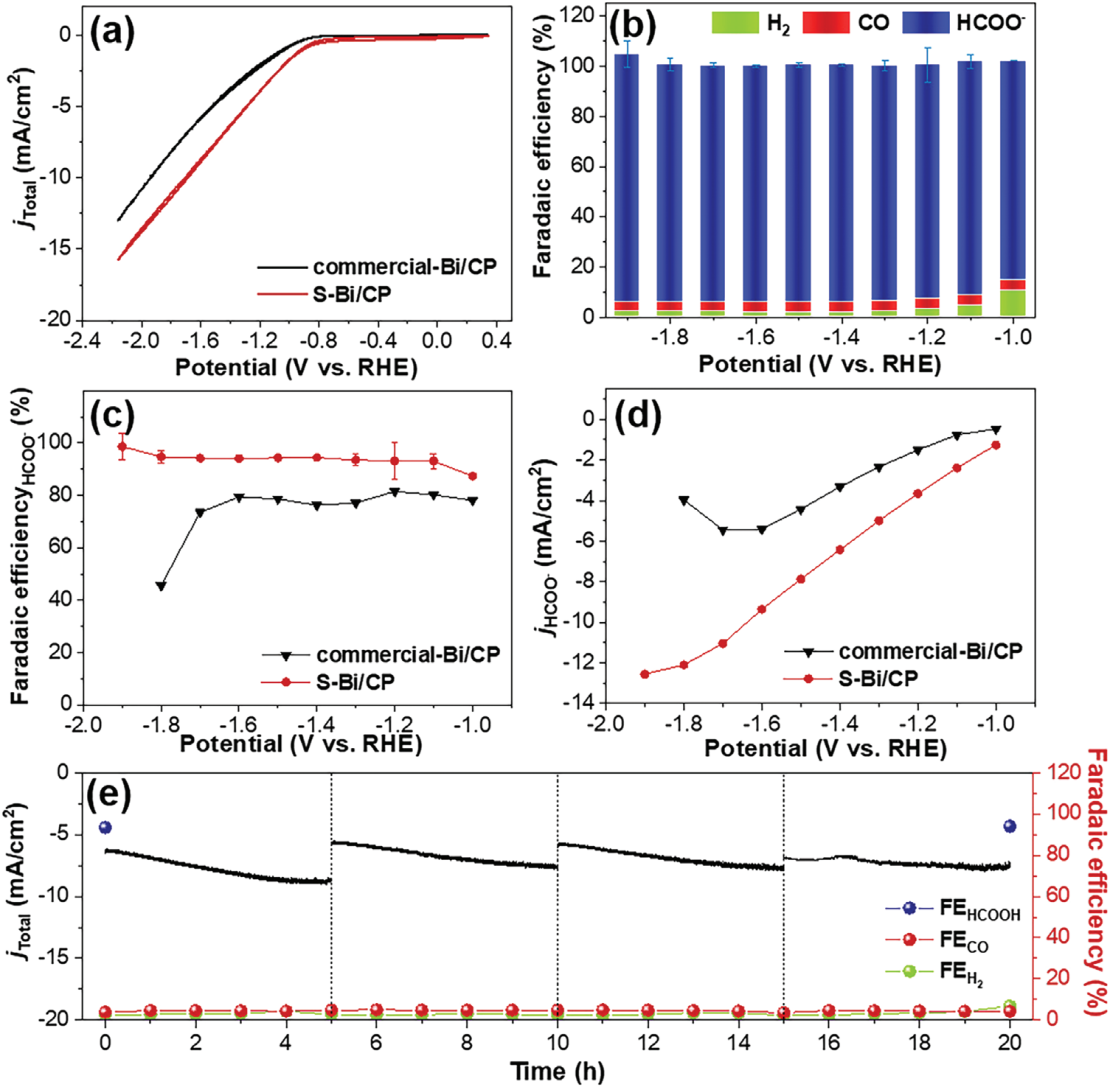
E‐CO_2_RR efficiency of S‐Bi/CP and commercial‐Bi/CP in 0.1 m KHCO_3_. a) CVs of S‐Bi/CP (red) and commercial‐Bi/CP (black) in CO_2_‐saturated electrolyte (scan rate: 10 mV s^−1^). b) Faradaic efficiency of S‐Bi/CP (blue: HCOO^−^, red: CO, green: H_2_) depending on applied potentials. c) Faradaic efficiency and d) partial current density of HCOO^−^ using S‐Bi/CP (red) and commercial‐Bi/CP (black). e) Chronoamperometric measurement and Faradaic efficiency of S‐Bi/CP at an applied potential of −1.4 V versus RHE over 20 h.

E‐CO_2_RR was performed using a three‐electrode H‐type cell with two compartments separated by a proton exchange membrane. The electrolyte comprised aqueous 0.1 m KHCO_3_, which was purged with CO_2_ during the E‐CO_2_RR process. A graphite rod served as the counter electrode, while a saturated calomel electrode (SCE) was used as the reference electrode. The electrocatalytic activity was preliminarily evaluated using cyclic voltammetry (CV) at a scan rate of 10 mV s^−1^ under CO_2_ or Ar atmosphere. The generated current was significantly higher under CO_2_‐saturated conditions than under Ar‐saturated conditions, indicating that the S‐Bi/CP electrode had higher catalytic activity for the CO_2_RR than for the HER (Figure S8, Supporting Information). Commercial‐Bi particles were subsequently utilized in conjunction with carbon paper to create a commercial‐Bi/CP electrode for comparison. As shown in **Figure** **2**a, the current density of the S‐Bi/CP electrode was enhanced (12.7 mA cm^−2^ at −1.9 V versus RHE) compared with that of commercial‐Bi/CP (9.8 mA cm^−2^).

The electrocatalytic selectivity of the S‐Bi/CP electrode was further investigated using chronoamperometry at constant applied potentials (Figure S9, Supporting Information). The gaseous products were analyzed by gas chromatography in real‐time, and the liquid products were characterized by proton nuclear magnetic resonance (^1^H NMR). In this specific reaction, the main product is HCOO^−^, whereas H_2_ and CO were negligible (Figure 2b). The S‐Bi/CP electrode generated HCOO^−^ with high selectivity over a wide potential range. The Faradaic efficiency of the S‐Bi/CP electrode for HCOO^−^ production exceeded 93% within the range of −1.1 to −1.9 V versus RHE and reached 94.4% at a potential of −1.9 V versus RHE. In contrast, the Faradaic efficiency of commercial‐Bi in the same potential range reached 81.5% at −1.2 V versus RHE, but the Faradaic efficiency decreased sharply at far negative applied potential (Figure 2c). The partial current densities of HCOO^−^ (*j*_HCOO_ *^−^)* are summarized in Figure 2d. Compared to the commercial‐Bi/CP electrode, the S‐Bi/CP electrode exhibited high efficiency in generating HCOO^−^ overall potential ranges. Many studies have reported selective HCOO^−^ generation in a narrow potential range; however, it is not easy to achieve good selectivity over such a wide potential range (Table S1, Supporting Information). A probable explanation for this is that S‐Bi contains numerous vacant sites resulting from the phase transition of Bi_2_S_3_, making the CO_2_RR activity of S‐Bi/CP potentially superior to that of commercial‐Bi/CP. This may be attributed to the high stability of adsorbed OCHO* on the S vacancies, which led to high activity and HCOO^−^ selectivity.

To evaluate material defects, Rietveld refinement was conducted using Bi particles (S‐Bi particles) synthesized via the chemical reduction of Bi_2_S_3_ nanorods (Figures S10,S11 and Table S2, Supporting Information). Small crystalline domains and lattice deformation, mainly caused by defects such as dislocations and point defects, can contribute to the broadening of X‐ray powder diffraction profiles and the shift in the peak positions. For this reason, the Rietveld refinement was carried out using TOPAS‐academic software to obtain more quantitative information from the X‐ray powder diffraction data, as shown in Figure S10 (Supporting Information).^[^ ^45^ ^]^ Of the refined parameters obtained from the Rietveld refinement, the site occupancy factor (SOF) describes the probability of finding the atom at a specific crystallographic site for any particular system. The value ranges from nearly 0 to a maximum of 1, depending on the Rietveld refinement programs. If each site and multiple sites are entirely filled, the SOF of that site is 1 (100%), and if there are some vacancies, i.e., due to such defects, the SOF is <1, meaning <100%. When the SOF is compared to the Bi‐atomic site (Wyckoff position 6c) for bare‐Bi and S‐Bi samples, the SOF of the S‐Bi particles (≈0.981(2)) was <1. In contrast, the SOF of the bare‐Bi particles is close to 1. In addition, a slight shift in the peak position was observed for the S‐Bi particles when compared to its corresponding diffraction peak in the bare‐Bi particles, as shown in Figure S11 (Supporting Information): the refined lattice parameters of the S‐Bi sample (a (= b) = 4.5412(2) Å, c = 11.8529(5) Å) were lower than those of the bare‐Bi sample (a (= b) = 4.5439(1) Å, c = 11.8564(4) Å). These results indicate that the S‐Bi sample has more defects than the bare‐Bi sample, although there is the presence of defects in both samples.

To further investigate the origin of the high HCOO^−^ Faradaic efficiency, we conducted comparative experiments using an unreduced Bi_2_S_3_/CP electrode (Figure S12, Supporting Information). However, in situ Raman analysis indicated that the initial Bi_2_S_3_ readily transforms into metallic Bi during the initial electrochemical reaction when the applied potential exceeds −0.1 V versus RHE (Figure 1i). Since the CO_2_ reduction onset potential is ≈−0.7 V versus RHE, the actual catalyst participating in the CO_2_ reduction reaction is primarily metallic Bi, which contains only trace amounts of S (≈1 wt.%, Figure S12, Supporting Information). The Bi_2_S_3_/CP electrode, whose actual state is 1 wt.% S‐doped Bi/CP, exhibited similar catalytic activity to S‐Bi/CP, but slightly negatively influenced catalytic selectivity (Figure S12d, Supporting Information). The Faradaic efficiency for HCOO^−^ production was ≈90% over the entire potential range. However, the HCOO^−^ Faradaic efficiency decreased to 61% at −1.0 V versus RHE. An augmentation in S content does not enhance catalytic activity or selectivity. Specific details are further studied in Section 2.3 (Theoretical Investigation).

To evaluate the stability of the S‐Bi/CP electrode, chronoamperometry was performed at −1.4 V versus RHE (Figure 2e). The current density of the S‐Bi/CP electrode maintained a steady‐state value for 20 h (with electrolyte replacement every 5 h), and the HCOO^−^ selectivity remained consistently above 93%. Additionally, even under high overpotential at −1.9 V, the stability of the S‐Bi/CP electrode persisted, with a Faradaic efficiency for HCOO^−^ exceeding 93% throughout successive E‐CO_2_RR (Figure S13, Supporting Information). These results indicate the robustness of the S‐Bi/CP electrode for CO_2_ reduction.

To investigate the structure and element distribution of the S‐Bi catalyst during CO_2_ reduction, additional characterizations (XRD, SEM‐EDS, and ICP‐OES) were conducted after the stability test. XRD analysis revealed that the phase of S‐Bi remained largely unchanged before and after electrochemical CO_2_ reduction (Figure S14, Supporting Information). SEM‐EDS confirmed consistent elemental distribution, with S content remaining at 0.4 wt.%, consistent with the initial state (Figure S6a, Supporting Information). Furthermore, ICP‐OES analysis confirmed the S content to be ≈0.66 wt.% (Figure S6b, Supporting Information). These results indicate that S‐Bi remains stable even when exposed to prolonged reaction conditions. Consequently, the catalytic activity of the S‐Bi/CP electrode remained consistent, with a Faradaic efficiency for HCOO^−^ exceeding 93% throughout consecutive E‐CO_2_RR, indicating that the S‐Bi electrode maintained its initial state.

### 2.3 Theoretical Investigation of CO_2_ Reduction Reaction

**Figure 3:**
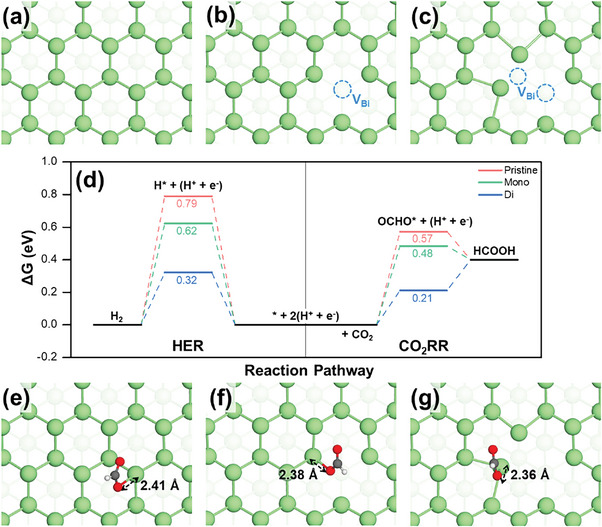
DFT‐optimized structures of a) pristine Bi surface, and defective Bi surfaces with b) mono‐vacancy and c) di‐vacancy. The Bi vacancy site (V_Bi_) is highlighted by a blue dashed circle. d) Free‐energy diagrams for CO_2_RR to generate formate and HER on the pristine Bi surface (pink line) and the defective surfaces (green line for mono‐vacancy surface and blue line for di‐vacancy surface). OCHO adsorption structures of e) pristine Bi surface, and defective Bi surfaces with f) mono‐vacancy and g) di‐vacancy. The green, red, gray, and white balls represent Bi, O, C, and H, respectively.

To determine the underlying reasons for the enhanced activity and selectivity of S‐Bi/CP compared to those of commercial‐Bi/CP in producing HCOOH, a comprehensive analysis using density functional theory (DFT) calculations was conducted. S‐Bi/CP derived from the electrochemical reduction of Bi_2_S_3_ is expected to possess numerous defects, unlike pristine Bi. Therefore, the DFT calculations covered three different models: a pristine Bi surface and defective Bi surfaces with mono‐(one) and di‐(two) vacancies, as shown in **Figure** **3**a–c. These models allowed an in‐depth exploration of the effects of defects on the CO_2_RR. Additional simulation details are provided in Computational Details.

The reduction of CO_2_ to HCOOH involves two elementary reaction steps, as represented by Equations (1 and 2):

(1) $$CO_{2}\left(g\right)+\left[H^{+}+e^{-}\right]+*\to \mathrm{OCHO}*$$

(2) $$\mathrm{OCHO}*+\left[H^{+}+e^{-}\right]\to *+\mathrm{HCOOH}\left(g\right)$$

where * and OCHO* denote the bare surface and the OCHO molecule adsorbed on the surface, respectively. Figure 3d shows the DFT‐computed free energy diagrams for the entire CO_2_RR compared with the HER, a competitive reaction in an aqueous electrolyte environment. These diagrams identify the rate‐determining step (RDS) for both reactions on all three Bi surfaces: conversion of CO_2_ to OCHO* for the CO_2_RR and the hydrogen adsorption (H*) for the HER. They also show how the vacancy sites change the overall reaction energy profiles. Increasing the number of vacancies strengthened the adsorption of the key intermediates (OCHO* for the CO_2_RR and H* for the HER) controlling the RDS, thereby reducing the free energy required for the reaction. Notably, for the CO_2_RR, the reaction‐free energy for RDS (∆*G*_RDS_) decreased from 0.57 eV on the pristine surface to 0.48 eV on the mono‐vacancy surface, and further to 0.21 eV on the di‐vacancy surface, indicating a significant reduction in the overpotential as the number of vacancies increased. The adsorption configuration of OCHO on each surface is shown in Figure 3e–g, with a detailed side view presented in Figure S15 (Supporting Information). The bond lengths (*d*_O‐Bi_) between the O atom of OCHO and the Bi atom of the surface decreased with increasing adsorption strength: 2.41 Å for the pristine surface, 2.38 Å for the mono‐vacancy surface, and 2.36 Å for the di‐vacancy surface. Similar trends were observed for the HER. As the number of vacancies increased, the ∆*G*_RDS_ declined from 0.79 eV on the pristine surface to 0.62 and 0.32 eV for the mono‐ and di‐vacancy surfaces, respectively. Notably, however, the ∆*G*_RDS_ for the HER remained significantly higher than that for the CO_2_RR, demonstrating better selectivity for the CO_2_RR over the HER on all three surface models.

We also extended our investigation to include CO_2_RR on both S‐doped pristine and defective Bi surfaces, as detailed in Figure S16 (Supporting Information). We examined S doping across different scenarios: on a pristine Bi surface (S‐Bi), and on defective surfaces with S atoms bonded to two and three Bi atoms (S‐V(S) and S‐V(Bi), respectively). These analyses revealed that S‐doped surfaces exhibit higher reaction‐free energies than their defect‐only counterparts. This highlights the significant impact of surface defects over S doping on CO_2_RR efficiency, emphasizing the importance of surface configurations in influencing catalytic activity.

**Figure 4:**
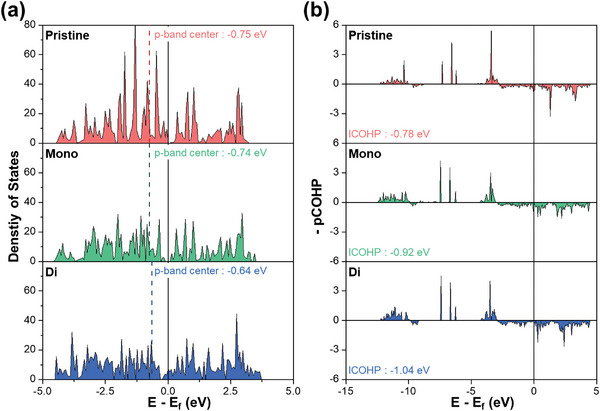
Electronic structure and bonding analysis. a) Density of states (DOS) for pristine Bi surface (top), mono‐vacancy Bi surface (middle), and di‐vacancy Bi surface (bottom). b) Projected crystal orbital Hamilton population (pCOHP) analysis depicting the bonding characteristics of OCHO when adsorbed on each Bi surface.

To investigate the impact of vacancies on the electronic structure and to verify the origin of the increased adsorption strength of OCHO, a key intermediate in the CO_2_RR, the density of states (DOS) was analyzed for each surface model. The formation of vacancies led to a reduction in the number of electrons, particularly near the Bi surface, causing a decrease in the orbital overlap between the Bi atoms and an upshift of the *p*‐band, as depicted in **Figure** **4**a. The *p*‐band center of the di‐vacancy surface was up‐shifted to −0.64 eV, representing a shift of 0.11 eV compared to that of the pristine surface (−0.75 eV). These distinctive *p*‐orbital characteristics near the vacancies on the Bi surface create an active site with increased affinity for adsorbates.

To further substantiate the role of the *p*‐orbital of the active site in adsorption, a projected crystal orbital Hamilton population (pCOHP) analysis was performed,^[^ ^46^ ^]^ focusing specifically on the interaction between the O atom of OCHO and the active Bi atom on the surface. In Figure 4b, the upper panel (‐pCOHP > 0) displays the bonding states, whereas the lower panel (‐pCOHP < 0) shows the anti‐bonding states. Figure 4b demonstrates that the bonding states between the O atom of OCHO and the Bi atom of the surface increased with the number of vacancies. The integrated crystal orbital Hamilton population (ICOHP) values, which indicate the bond strength (more negative values imply stronger bonds), confirm that the bond between the O atom of OCHO and the Bi atom was stronger on the di‐vacancy surface compared to that on the other surfaces. This finding is consistent with the DOS results. The ICOHP value for the defective Bi surface with the di‐vacancy is −1.04 eV, which is 0.12 and 0.26 eV lower than that of the mono‐vacancy surface and pristine surface, respectively.

The DFT calculations showed the interplay between the surface defects and enhanced adsorption of OCHO, leading to a reduced free‐energy barrier for the CO_2_RR. These theoretical findings, corroborated by experimental data, underscore the pivotal role of surface defects in optimizing the catalytic efficiency of S‐Bi/CP for HCOOH production via CO_2_ reduction.

### 2.4 Synthesis and Characterization of the Si Nanowires (SiNWs) and S‐Bi/SiNWs

**Figure 5:**
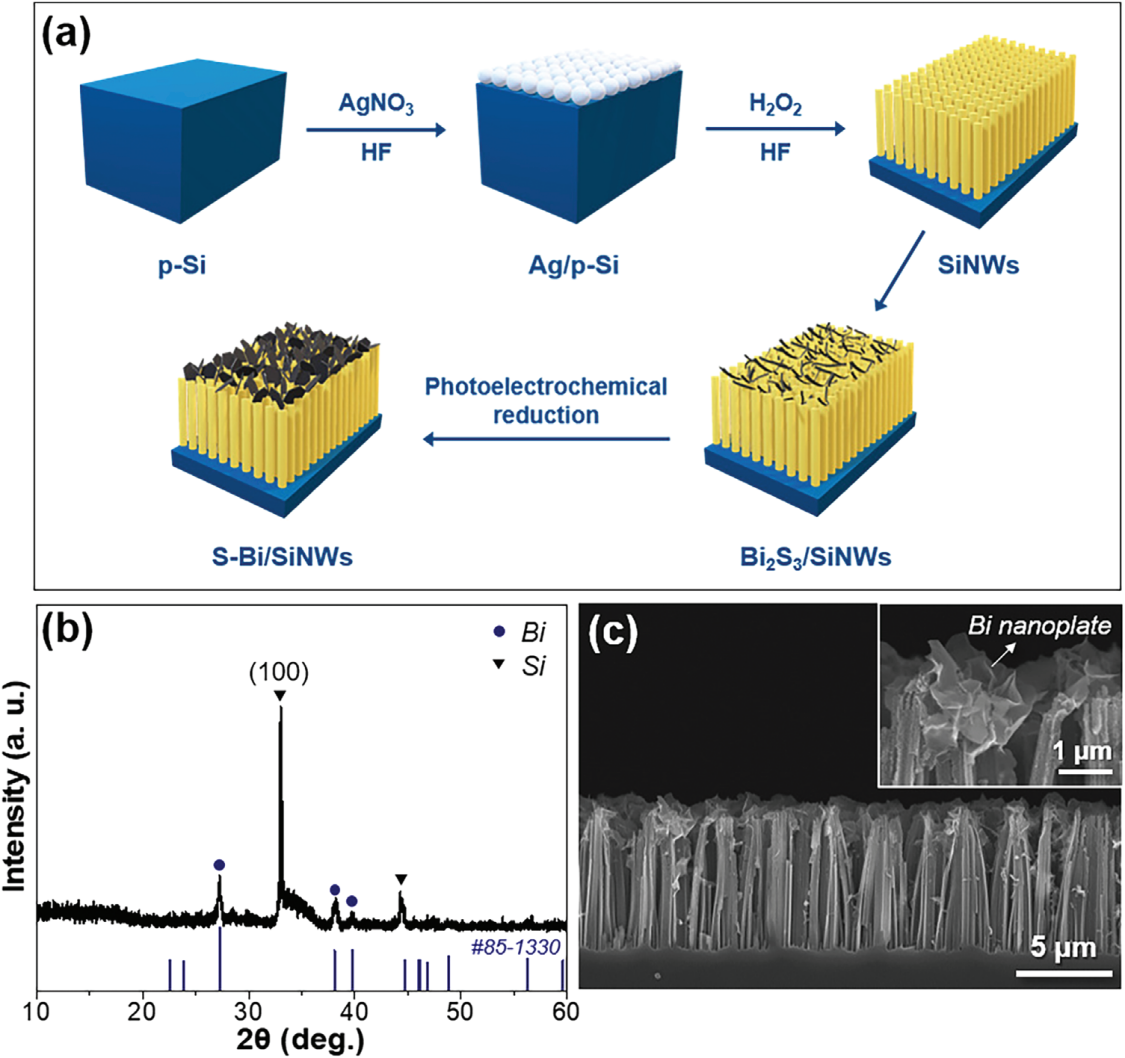
a) Schematic of the fabrication of the S‐Bi/SiNWs photocathode. b) XRD pattern and c) cross‐sectional SEM image of S‐Bi/SiNWs at low and (inset) high magnifications.

The SiNWs were fabricated using metal‐induced electroless etching on a p‐type Si (100) wafer according to the procedure illustrated in **Figure** **5**a.^[^ ^47^ ^]^ The length of the SiNWs gradually increased with the etching time (≈3, 6, and 12 µm at 10, 20, and 35 min, respectively), with an average diameter of 150 nm (Figure S17, Supporting Information). The total reflectance spectra, measured using an UV–vis spectrophotometer with an integrating sphere, showed that the planar Si suffered from strong reflectance losses across the entire spectral range due to the large gap of the refractive indices (*n*) of air (*n* = 1.06) and Si (*n* = 4), whereas the SiNWs could reduce the reflectance of incident light, enabling more efficient light absorption due to the superior light scattering properties of the SiNWs (Figure S18, Supporting Information).^[^ ^48^ ^]^ Linear sweep voltammetry (LSV) was used to investigate the PEC response of both planar Si and SiNWs electrodes in CO_2_‐saturated 0.1 m KHCO_3_ electrolyte under simulated solar irradiation (AM 1.5G) (Figure S19, Supporting Information). The Si electrodes produced only H_2_, even in a CO_2_‐saturated 0.1 m KHCO_3_ electrolyte. In general, Si nanostructures demonstrate similar PEC activities regardless of the surface shape. In multi‐electron reactions such as HER and CO_2_RR, enlarging the junction area of semiconductors such as nanowires is expected to boost the photocurrent by reducing the travel distance of the minority carriers. However, this area augmentation was accompanied by surface recombination, predominantly impacting the current, thereby resulting in a reduced open‐circuit potential and thus lower photocurrents.^[^ ^49^ ^]^ The photocurrents of planar Si and the SiNWs were similar, irrespective of the light absorption (Figure S19, Supporting Information). A representative SiNW sample with a length of 6 µm was characterized in detail for PEC reactions.

The Bi electrocatalyst was deposited on the SiNWs by drop‐casting Bi_2_S_3_ on the SiNWs, followed by photoelectrochemical reduction (Figure 5a). The Bi_2_S_3_ phase was transformed to S‐Bi on the SiNWs by applying a potential of −1.4 V for 1 h in 0.1 m KHCO_3_ under simulated solar irradiation (AM 1.5G). Figure 5b shows the XRD patterns of the transformed metallic Bi and the SiNWs electrode. Bi nanoplates were uniformly deposited on the SiNWs (Figure 5c). The XRD results show that Bi_2_S_3_ was completely reduced to Bi; however, the remaining S (≈0.3 wt.%) was also observed in the TEM‐EDS analysis (Figure S20, Supporting Information). The obtained S‐Bi/SiNWs photocathode was utilized in the PEC‐CO_2_RR.

### 2.5 Photoelectrochemical CO_2_ Reduction Reaction (PEC‐CO_2_RR) of S‐Bi/SiNWs

The PEC‐CO_2_RR with a p‐Si photocathode can significantly decrease the overpotential required for CO_2_RR. However, since a pure p‐Si photocathode exclusively produces H_2_ (Figure S21, Supporting Information), appropriate co‐catalysts are necessary to enable selective CO_2_RR. Nevertheless, it cannot be guaranteed that an electrocatalyst efficient for E‐CO_2_RR will perform equally effectively as a co‐catalyst on the semiconductor surface.^[^ ^50^ ^]^ To elucidate the interaction sites between the Si surface and the co‐catalyst, a perfectly selective electrocatalyst is imperative. Due to the superior catalytic activity and selectivity toward HCOO^−^ production in the E‐CO_2_RR exhibited by the resulting S‐Bi nanoplates, they were deposited on SiNWs to fabricate an S‐Bi/SiNWs photocathode, facilitating the mechanistic understanding of PEC‐CO_2_RR. Given that increasing S content does not enhance catalytic activity or selectivity, the PEC‐CO_2_RR experiment was conducted using a fully reduced S‐Bi on the SiNWs electrode.

**Figure 6:**
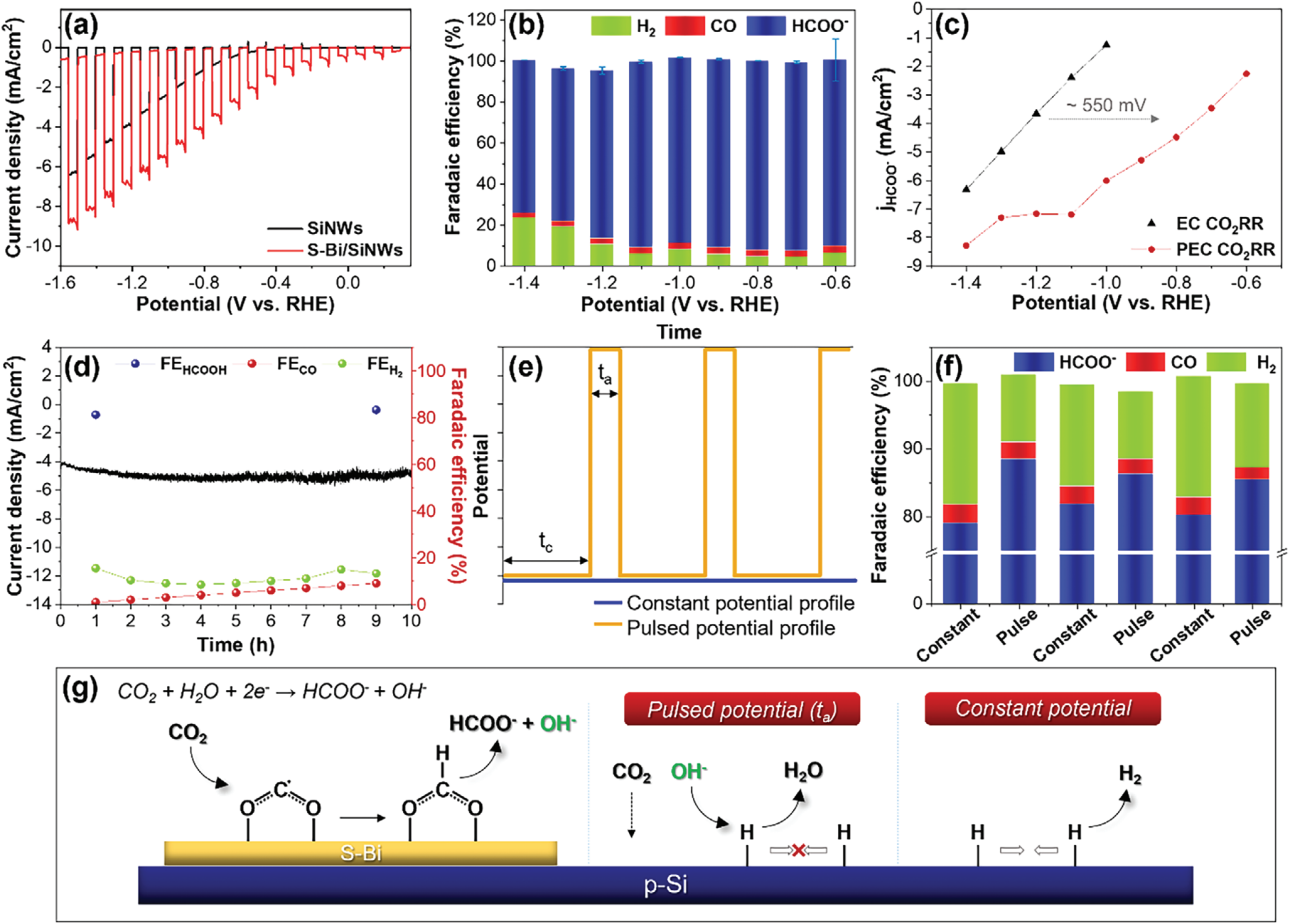
PEC‐CO_2_RR efficiency of S‐Bi/SiNWs and SiNWs in 0.1 m KHCO_3_ under light irradiation (light intensity: 100 mW cm^−2^). a) LSVs in CO_2_‐saturated electrolyte (scan rate: 10 mV s^−1^). b) Faradaic efficiency (blue: HCOO^−^, red: CO, green: H_2_) of S‐Bi/SiNWs depending on applied potentials. c) Comparison of the partial current density of HCOO^−^ for S‐Bi/CP (black) and S‐Bi/SiNWs (red). d) Stability of S‐Bi/SiNWs at −0.8 V versus RHE and the corresponding Faradaic efficiency. e) Schematic of applied constant potential (blue) and pulsed potential (yellow). f) Consecutive Faradaic efficiency (blue: HCOO^−^, red: CO, green: H_2_) of S‐Bi/SiNWs during alternating pulsed potential cycles (*E*_−1.4 V_ = 3 s, *E_0_*_.1 V_ = 1 s) and constant potential (*E*_−1.4 V_) measurements. g) Schematic of the proposed CO_2_ reduction mechanism during the pulsed potential and constant potential electrolysis.

The PEC‐CO_2_RR was evaluated using the prepared S‐Bi/SiNWs photocathode under simulated solar irradiation (light intensity: 100 mW cm^−2^). The PEC performance was investigated using LSV at a scan rate of 10 mV s^−1^ with chopped light irradiation in a CO_2_‐saturated 0.1 m KHCO_3_ electrolyte. The S‐Bi/SiNWs exhibited significantly higher photocurrents than the SiNWs over the entire potential range (**Figure** **6**a). The photocurrent density of the S‐Bi/SiNWs was 4.1 mA cm^−2^, whereas that of SiNWs was 1.3 mA cm^−2^ at −0.8 V versus RHE. The onset potential was positively shifted by ≈400 mV compared to that of the SiNWs. In addition, the PEC activity of the S‐Bi/SiNWs photocathode was considerably higher under CO_2_‐saturated conditions than under Ar (Figure S22, Supporting Information). The catalytic selectivity of the S‐Bi/SiNWs photocathode was investigated using chronoamperometry at a constant applied potential under light irradiation. During the PEC‐CO_2_RR, S‐Bi functioned as a catalyst for CO_2_ reduction rather than H_2_ generation on the SiNWs, and this selectivity matched well with the E‐CO_2_RR results for S‐Bi/CP (Figure 2). The major product obtained with the S‐Bi/SiNWs was HCOO^−^ (over 90%) in the range of −0.6 to −1.1 V versus RHE, with H_2_ being a minor product, whereas CO was produced in negligible quantities. The maximum Faradaic efficiency for HCOO^−^ of the S‐Bi/SiNWs photocathode was 92.3% at −0.8 V versus RHE (Figure 6b; Figure S23, Supporting Information). When a potential exceeding −1.2 V versus RHE was applied, the H_2_ production increased (the related mechanism is discussed in the following section). By contrast, SiNWs without the co‐catalyst produced only H_2_ at all applied potentials. And when the pH decreases, H_2_ production increases, showing a pH‐dependent property, even in CO_2_‐saturated conditions (Figure S21, Supporting Information). Thus, the SiNWs surface primarily contributed to the production of H_2_, whereas the S‐Bi surface was mainly responsible for generating HCOO^−^. Even with a pH value of 3, high selectivity in formate production was observed, confirming the predominance of CO_2_RR on the S‐Bi surface (Figure S24, Supporting Information). Although the Si surface was exposed to water, the fabricated S‐Bi/SiNWs photocathode exhibited high selectivity toward HCOO^−^ in the low overpotential range. These observations suggest that S‐Bi rapidly extracts photogenerated electrons from the Si NWs and facilitates CO_2_ reduction on its surface. In other words, considering the overpotential needed for the conversion of CO_2_ to HCOO^−^, the PEC system using the S‐Bi/SiNWs photocathode required a lower overpotential compared to the EC system using S‐Bi/CP (Figure 6c).

To elucidate the influence of the Si photocathodes, the PEC performance of S‐Bi on a Si wafer (planar Si substrate, Figure S25, Supporting Information) was further investigated. The planar Si wafer photocathode exhibited a low photocurrent density and limited stability when S‐Bi was deposited. The S‐Bi co‐catalyst rapidly detached from the Si wafer during the PEC‐CO_2_RR owing to the slippery nature of the single‐crystalline Si surface, leading to a significant decrease in the activity and selectivity toward HCOO^−^. Considering the requirement for long‐term operation with a high current density, SiNWs are highly suitable for co‐catalyst deposition, as they can maintain the stable state of the S‐Bi/SiNWs composite over extended periods. The durability of the catalysts was evaluated using chronoamperometry under light irradiation at −0.8 V versus RHE (Figure 6d). The photocurrent density of the S‐Bi/SiNWs maintained a steady‐state value for 10 h, and the Faradaic efficiency of the S‐Bi/SiNWs for HCOO^−^ remained consistently above 90% throughout the reaction. These results indicate that the photocathode assembled using the S‐Bi co‐catalyst on the SiNWs exhibited both high performance and stability for the PEC‐CO_2_RR.

In the E‐CO_2_RR employing S‐Bi, a negligible quantity of H_2_ was noted at −1.3 V versus RHE. In contrast, higher production of H_2_ (≈20%) was detected at the same potential in the PEC‐CO_2_RR. Despite employing the same potential, both the CO_2_RR and HER processes occurred concurrently on the S‐Bi/SiNWs at −1.3 V versus RHE. It is imperative to investigate whether the Bi or Si surfaces function as the active sites for the undesired HER during the PEC reaction. Pulsed electrolysis was used to improve the product selectivity within the negative potential range above −1.3 V versus RHE by modulating the double‐layer environment during the PEC‐CO_2_RR (Figure 6e). The chosen potentials for pulsed electrolysis were 0.1 and −1.4 V versus RHE. Throughout this process, the cathodic and anodic pulse times (*t_c_* and *t_a_*) were sustained at 3 s at −1.4 V and 1 s at 0.1 V versus RHE under light irradiation, respectively. For both constant and pulsed electrolysis, the electrical charges remained consistent. Interestingly, when pulsed electrolysis was applied to the S‐Bi/SiNWs, a remarkable increase in the HCOO^−^ selectivity (>5%) was observed compared to that under constant electrolysis (Figure 6f). Because HCOO^−^ was the sole product generated when S‐Bi was employed in the E‐CO_2_RR at the same potential (Figure 2b), H_2_ generation is postulated to occur on the exposed Si surface. This suggests that pulsed electrolysis may impede H_2_ production on the Si surface within the S‐Bi/SiNWs photocathode. To summarize the reaction process, the OH^−^ ions generated during the pulsed PEC‐CO_2_RR caused a deceleration in the dimerization of adsorbed H during the anodic pulse (0.1 V), subsequently suppressing the HER on the Si surface (Figure 6g). This, in turn, led to CO_2_ diffusion and an increase in the Faradaic efficiency for HCOO^−^ production within the S‐Bi/SiNWs photocathode. This pulsed photoelectrolysis approach could potentially serve as a novel method for further enhancing the Faradaic efficiency of the PEC‐CO_2_RR system.

## 3 Conclusion

This study presented a novel approach to convert Bi_2_S_3_ nanorods into metallic Bi nanoplates, resulting in catalysts that exhibited strong adhesion to substrates and remarkable catalytic activity for converting CO_2_‐to‐HCOO^−^ in both electrochemical and photoelectrochemical systems. The resulting S‐Bi nanoplates, when deposited on a carbon paper substrate, displayed superior catalytic performance in the E‐CO_2_RR, achieving a Faradaic efficiency exceeding 93% across a broad potential range. This exceptional efficiency stood out as previous studies had reported selective HCOO^−^ generation within narrower potential windows. The study delved into the role of defect states within the Bi catalyst, derived from Bi_2_S_3_, in enhancing catalytic efficiency. DFT calculations revealed that these defect states played a crucial role in stabilizing intermediates, thus lowering the free‐energy barrier for the CO_2_ reduction process. Moreover, when the S‐Bi catalyst was applied to a photocathode composed of S‐Bi on SiNWs, it exhibited high activity and selectivity in HCOO^−^ production under simulated solar light irradiation. Remarkably, the electrocatalytic selectivity of the S‐Bi catalyst in the E‐CO_2_RR mirrored its performance in the PEC‐CO_2_RR with the S‐Bi/SiNWs photocathode, even within the low overpotential range. However, a notably higher production of H_2_ (≈20%) was observed at the large over‐potential range in the PEC‐CO_2_RR. To address this, pulsed electrolysis techniques were employed, effectively suppressing HER and enhancing the Faradaic efficiency of HCOO^−^. These findings highlighted the potential of S‐Bi as a versatile catalyst for CO_2_ reduction reactions, offering insights into optimizing reaction mechanisms and improving product selectivity, particularly when combined with co‐catalysts for Si photocathode in the PEC system.

## 4 Experimental Section

### Synthesis of Bi_2_S_3_

Powdered Bi_2_S_3_ nanorods were synthesized using a hydrothermal reaction. Bi_2_O_3_ powder, thiourea (7.9 mmol), and hydrochloric acid (2 m, 100 µL) were added to deionized water (30 mL). The solution was then transferred to an autoclave and heated in an electric oven at 160 °C for 6 h. The resulting Bi_2_S_3_ nanorods were centrifuged, washed multiple times with ethanol, and dried under ambient atmosphere at 60 °C. The prepared Bi_2_S_3_ was subsequently reduced to Bi on the CP (S‐Bi/CP) and SiNWs (S‐Bi/SiNWs) for utilization in the E‐CO_2_RR and PEC‐CO_2_RR.

### Synthesis of Bare Bi

The bare Bi sample was synthesized based on a simple reduction reaction using Bi(NO_3_)_3_·5H_2_O as the Bi precursor and NaBH_4_ as the reducing agent. Briefly, a slurry of Bi(NO_3_)_3_·5H_2_O (1.5 mmol), NaBH_4_ (60 mmol), and 2‐ethoxyethanol (50 mL) was prepared in a 100 mL flask, which was then submerged in a preheated oil‐bath at 120 °C. The flask was then connected to a bubbler for gas exchange. The slurry was stirred and maintained at this temperature for 30 min, and the resulting black mixture was cooled to room temperature. The obtained suspension was centrifuged, and the residue was washed with ethanol a dried under ambient atmosphere at 60 °C.

### Computational Details

Spin‐polarized density functional theory (DFT) calculations were performed using the Vienna Ab initio Simulation Package.^[^ ^51^ ^]^ In these calculations, projector‐augmented wave (PAW)^[^ ^52^ ^]^ pseudopotentials and the Perdew–Burke–Ernzerhof exchange‐correlation functional^[^ ^53^ ^]^ were employed. For a more precise description of the van der Waals interactions, Grimme's D3 dispersion correction^[^ ^54^ ^]^ was included. The energy cutoff was set to 500 eV and Brillouin zone sampling was performed using a (10 × 10 × 4) k‐point grid for the bulk model and a (3 × 3 × 1) k‐point grid for the surface models.

A hexagonal Bi bulk crystal structure belonging to the R‐3m (166) space group was used as the computational model and aligned with the metallic Bi phase identified in the XRD results. The optimized lattice constants of the Bi bulk structure are *a* = 4.5657 Å and *c* = 11.7647 Å, which are in good agreement with a previous study.^[^ ^55^ ^]^ The Bi (001) surface was used as a model of the Bi surface as this is the most stable low‐index plane surface, consisting of six atomic layers with 54 Bi atoms. For a comprehensive analysis of the changes in the reaction‐free energy due to surface defects, the vacancy formation energy associated with the Bi vacancies located in the top, middle, and bottom layers (Figure S26a, Supporting Information) was calculated; the surface model with vacancies in the top layer was used, which resulted in maximum stability. A vacuum space of ≈15 Å along the *z*‐direction was included in all surface models to avoid artificial interactions between the periodic replicas. During the optimization of the surface models, the bottom three layers were fixed, whereas the top three layers and adsorbates were allowed to relax.

As in the previous DFT studies,^[^ ^56^, ^57^, ^58^ ^]^ the free energy of each reaction step was computed at 298.15 K by considering the zero‐point energy, entropy, and enthalpy. Additionally, the solvation effect was incorporated using the implicit solvation model available in VASPsol.^[^ ^59^, ^60^ ^]^ As shown in Figure S26b–d (Supporting Information), the possible adsorption sites on each surface model were extensively examined for OCHO adsorption. However, only the most stable OCHO‐adsorption configuration was considered in this study. The chemical bonding properties were analyzed by using the projected crystal orbital Hamilton population (COHP) analysis method available in the LOBSTER software package.^[^ ^61^ ^]^

## Conflict of Interest

The authors declare no conflict of interest.

## Author Contributions

A.M., Y.L., and D.S. contributed equally to this study. The study was designed by K.M.N., H.D.U., and H.S. Catalyst synthesis and characterization were conducted by A.M., D.S., S.P., W. K., D.H.N., H.L., and Y.‐I.K.Y.L. and J.S. performed the theoretical experiments. K.M.N. and the co‐authors contributed to writing the manuscript.

## Supporting information

Supporting Information

## Data Availability

The data that support the findings of this study are available from the corresponding author upon reasonable request.
