# Supplementary material for: Unlocking the Potential of Bi2S3‐Derived Bi Nanoplates: Enhanced Catalytic Activity and Selectivity in Electrochemical and Photoelectrochemical CO2 Reduction to Formate
Source: Adv Sci (Weinh). 2024 May 17;11(28):2400874. doi: 10.1002/advs.202400874 (PMC11267354; doi:10.1002/advs.202400874)
Supplement: Supplementary file 1 — Supporting Information [file ADVS-11-2400874-s001.pdf]

ADVANCED SCIENCE
Open Access

Supporting Information

for *Adv. Sci.*, DOI 10.1002/advs.202400874

Unlocking the Potential of Bi_2S_3-Derived Bi Nanoplates: Enhanced Catalytic Activity and Selectivity in Electrochemical and Photoelectrochemical CO_2 Reduction to Formate

Ahyeon Ma, Yongsoon Lee, Dongho Seo, Jiyoon Kim, Soohyeok Park, Jihoon Son, Woosuck Kwon, Dae-Hyun Nam, Hyosung Lee, Yong-Il Kim, Han-Don Um, Hyeyoung Shin* and Ki Min Nam**

Supporting Information

Unlocking the Potential of Bi_2S_3-Derived Bi Nanoplates: Enhanced Catalytic Activity and Selectivity in Electrochemical and Photoelectrochemical CO_2 Reduction to Formate

Ahyeon Ma,[†] Yongsoon Lee,[†] Dongho Seo,[†] Jiyoon Kim, Soohyeok Park, Jihoon Son, Woosuck Kwon, Dae-Hyun Nam, Hyosung Lee, Yong-Il Kim, Han-Don Um, Hyeyoung Shin,* and Ki Min Nam**

A. Ma, D. Seo, J. Kim, K.M. Nam
Department of Chemistry and Institute for Future Earth, Pusan National University, Geumjeong-gu, Busan 46241, Republic of Korea
kimin.nam@pusan.ac.kr
Y. Lee, J. Son, H. Shin
Graduate School of Energy Science and Technology (GEST), Chungnam National University, Daejeon 34134, Republic of Korea
shinhy@cnu.ac.kr
S. Park, H.-D. Um
Department of Chemical Engineering, Kangwon National University, Chuncheon, Gangwon-do 24341, Republic of Korea
handon@kangwon.ac.kr
W. Kwon, D.-H. Nam
Department of Energy Science and Engineering, Daegu Gyeongbuk Institute of Science and Technology (DGIST), Daegu 42988, Republic of Korea
H. Lee, Y. -I. Kim
Korea Research Institute of Standards and Science (KRISS), 267 Gajeong, Yuseong, Daejeon, 34113, Republic of Korea
Department of Measurement Engineering, University of Science and Technology, 217, Gajeong, Yuseong, Daejeon 34113, Republic of Korea

[†]These authors contributed equally

*Corresponding Authors

E-mail: kimin.nam@pusan.ac.kr, handon@kangwon.ac.kr, shinhy@cnu.ac.kr

Experimental Section

Materials. $Bi(NO_3)_3 \cdot 5H_2O$ (99.999%, Sigma-Aldrich) was used as a metal precursor salt. Thiourea (≥99%, Sigma-Aldrich) and hydrochloric acid (36.5%, JUNSEI) were used as received. Deionized water was used as the solvent in all the electrochemical experiments. Carbon paper (MGL 190, AvCarb) was used as a substrate. P-type 6" wafers (<100> oriented, 1-10 Ohm-cm, prime, double-sided polished) were used for fabrication.

Fabrication of *p*-Si nanowire array. The SiNWs electrode was prepared by metal-induced electroless etching on a Si(100) wafer (p-type, 1-10 Ω cm) according to the procedure illustrated in **Figure** 5(a). Ag particles were first precipitated onto a hydrogen-terminated Si wafer by electroless deposition in an aqueous solution of 4.8 M hydrofluoric acid (HF) and 0.01 M $AgNO_3$ at room temperature. After the deposition of the metal particles, the sample wafer was immersed in an oxidizing HF etching solution (4.8 M HF and 0.44 M H_2O_2) for 6 min, then copiously rinsed with deionized H_2O and dried at room temperature. The wafers were then cleaned in diluted HNO_3 to remove residual Ag particles. For the photoelectrochemical tests, a 250 nm thick aluminum layer was deposited via thermal evaporation to obtain an ohmic contact on the backside of the Si wafer. The Si wafers were cut into pieces (2.0 cm × 1.0 cm) and masked with polyimide tape and only a certain fraction of the area (1 cm^2) was exposed to the electrolyte.

Synthesis of Bi_2S_3. Powdered Bi_2S_3 nanorods were synthesized using a hydrothermal reaction. Bi_2O_3 powder, thiourea (7.9 mmol), and hydrochloric acid (2 M, 100 µL) were added to deionized water (30 mL). The solution was then transferred to an autoclave and heated in an electric oven at 160 °C for 6 h. The resulting Bi_2S_3 nanorods were centrifuged, washed multiple times with ethanol,

and dried under ambient atmosphere at 60 °C. The prepared Bi_2S_3 was subsequently reduced to Bi on the CP (S-Bi/CP) and SiNWs (S-Bi/SiNWs) for utilization in the E-CO_2RR and PEC-CO_2RR.

Synthesis of bare Bi. The bare Bi sample was synthesized based on a simple reduction reaction using $Bi(NO_3)_3 \cdot 5H_2O$ as the Bi precursor and $NaBH_4$ as the reducing agent. Briefly, a slurry of $Bi(NO_3)_3 \cdot 5H_2O$ (1.5 mmol), $NaBH_4$ (60 mmol), and 2-ethoxyethanol (50 mL) was prepared in a 100 mL flask, which was then submerged in a preheated oil-bath at 120 °C. The flask was then connected to a bubbler for gas exchange. The slurry was stirred and maintained at this temperature for 30 min, and the resulting black mixture was cooled to room temperature. The obtained suspension was centrifuged, and the residue was washed with ethanol a dried under ambient atmosphere at 60 °C.

Synthesis of S-Bi particles. Powdered S-Bi particles were synthesized by simple $NaBH_4$ treatment. Briefly, a slurry of Bi_2S_3 nanorods, $NaBH_4$ (60 mmol), and 2-ethoxyethanol (50 mL) was mixed in a 100 mL flask. The slurry was stirred and maintained at 25 °C for 30 min, and the resulting black mixture was cooled to room temperature. The obtained suspension was centrifuged, and the residue was washed with ethanol a dried under ambient atmosphere at 60 °C.

Preparation of working electrode. The electrodes were prepared using a drop-coating method. Carbon Paper (1 cm ×1 cm) and SiNWs (1 cm ×1 cm) were used as substrates. The steps in preparing the electrode are as follows: 10 mg catalyst was mixed with 300 μL of ethanol, 700 μL H_2O, and 100 μL Nafion under ultrasonic treatment for 30 min to form a homogeneous suspension. To deposit the Bi_2S_3 co-catalysts on the electrode, the Bi_2S_3 ink 20 μL was drop-casted onto the surface of the electrode. The working electrodes were then dried and stored.

Electrochemical performance. All electrochemical properties were measured using an electrochemical station (CHI604 Austin, TX, Ivium V89128 Vertex One). Electrochemical measurements were conducted in a three-electrode H-type cell separated using a Nafion 115 membrane. A graphite rod and a saturated calomel electrode (SCE) were used as the counter and reference electrodes, respectively. The electrolyte comprised aqueous 0.1 M $KHCO_3$ that was purged with CO_2 prior to testing. The flow rate of CO_2 was maintained at 10 sccm and the electrolyte was continuously stirred at 300 rpm during the measurements. All the applied potentials were recorded against the SCE reference electrode and then converted to the reversible hydrogen electrode (RHE) scale without ohmic resistance correction:

$$E \text{ (vs. RHE)} = E \text{ (vs. SCE)} + 0.241 + 0.0592 \text{ V} \times \text{pH}$$

The gaseous products were analyzed using a gas chromatograph (6500GC System; YL Instrument Co.) equipped with a Carboxen-1000 column, thermal conductivity detector, flame ionization detector, and methanizer, with Ar (99.999%) as the carrier gas. The liquid products were quantified by nuclear magnetic resonance (NMR) (Bruker 400 MHz), in which 0.45 mL catholyte was mixed with 0.05 mL of phenol (diluted to 30 mM by deuterated water) as an internal standard. Because the production of H_2, CO, and $HCOO^-$ is a two-electron process, the Faradaic efficiencies of H_2, CO, and $HCOO^-$ were estimated using the following equation:

$$\text{Faradaic efficiency} = 2\, F \times n_i \,/\, (I \times t)$$

where F is Faraday's constant (96485 C mol^{-1}), n_i is the number of moles of H_2, CO, or $HCOO^-$ (mol), I is the current (A), and t is the time (s).

Photoelectrochemical measurement. All photoelectrochemical properties were measured using an electrochemical station (CHI604 Austin, TX, Ivium V89128 Vertex One). Photoelectrochemical measurements were conducted in a three-electrode H-type cell separated using a Nafion 115 membrane. A graphite rod and a saturated calomel electrode (SCE) were used as the counter and reference electrodes, respectively. The electrolyte comprised aqueous 0.1 M $KHCO_3$ that was purged with CO_2 prior to testing. The flow rate of CO_2 was maintained at 10 sccm and the electrolyte was continuously stirred at 300 rpm during the measurements. The front side of the p-Si was illuminated with AM 1.5G light at 100 mW/cm^2. All the applied potentials were recorded against the SCE reference electrode and then converted to the reversible hydrogen electrode (RHE) scale without ohmic resistance correction:

$$E \text{ (vs. RHE)} = E \text{ (vs. SCE)} + 0.241 + 0.0592 \text{ V} \times \text{pH}$$

The gaseous products were analyzed using a gas chromatograph (6500GC System; YL Instrument Co.) equipped with a Carboxen-1000 column, thermal conductivity detector, flame ionization detector, and methanizer, with Ar (99.999%) as the carrier gas. The liquid products were quantified by nuclear magnetic resonance (NMR) (Bruker 400 MHz), in which 0.45 mL catholyte was mixed with 0.05 mL of phenol (diluted to 30 mM by deuterated water) as an internal standard. Because the production of H_2, CO, and $HCOO^-$ is a two-electron process, the Faradaic efficiencies of H_2, CO, and $HCOO^-$ were estimated using the following equation:

$$\text{Faradaic efficiency} = 2\, F \times n_i \,/\, (I \times t)$$

where F is Faraday's constant (96485 C mol^{-1}), n_i is the number of moles of H_2, CO, or $HCOO^-$ (mol), I is the current (A), and t is the time (s).

Characterization. The samples were characterized by X-ray diffraction (XRD, X'Pert) with Cu-Kα radiation (λ =1.5406 Å) at 60 kV over the 2θ range of 5–80°. The diffraction peaks of the crystalline phase were compared with those of standard compounds reported in the ICDD data files. XRD for Rietveld refinement was collected at the Korea Advanced Institute of Science and Technology (KAIST) Analysis Center for Research Advancement (KARA). The data was acquired using a Bragg-Brentano Smartlab (Rigaku, Japan) diffractometer with a 300 mm radius and a Cu anode (CuKα1) radiation. The data acquisition range was from 10° to 145° with a 0.01° step. The nanostructures and morphologies were examined by high-resolution scanning electron microscopy (HR-SEM, Zeiss SUPRA 40VP) at an accelerating voltage of 10 kV. Transmission electron microscopy (TEM; Thermo Fischer aberration-corrected SPECTRA 300 microscope) images of the colloidal solution were acquired using a carbon-coated copper grid (200 mesh, F/C-coated, Ted Pella Inc.). X-ray photoelectron spectroscopy (XPS) measurements were performed using a K-alpha spectrometer with an Al Kα X-ray source and a pass energy level of 40 eV.

In situ Raman analysis. *In-situ* Raman spectroscopy was conducted using an XploRA PLUS Raman spectrometer (HORIBA). An altered flow cell with a water immersion objective lens (60×) and 785 nm laser was employed. The CO_2 gas flow was maintained at 50 sccm using a mass flow controller (MFC). Platinum foil and Ag/AgCl electrode (saturated KCl solution) served as the counter and reference electrodes, respectively. Raman spectra were acquired with a 5 s acquisition time, accumulated five times.

Computational details. Spin-polarized density functional theory (DFT) calculations were performed using the Vienna Ab initio Simulation Package.[1] In these calculations, projector-

augmented wave (PAW)[2] pseudopotentials and the Perdew–Burke–Ernzerhof exchange-correlation functional[3] were employed. For a more precise description of the van der Waals interactions, Grimme's D3 dispersion correction[4] was included. The energy cutoff was set to 500 eV and Brillouin zone sampling was performed using a (4 × 4 × 4) k-point grid for the bulk model and a (4 × 4 × 1) k-point grid for the surface models.

A hexagonal Bi bulk crystal structure belonging to the R-3m (166) space group was used as the computational model and aligned with the metallic Bi phase identified in the XRD results. The optimized lattice constants of the Bi bulk structure are a = 4.5657 Å and c = 11.7647 Å, which are in good agreement with a previous study.[5] The Bi (001) surface was used as a model of the Bi surface as this is the most stable low-index plane surface, consisting of six atomic layers with 54 Bi atoms. For comprehensive analysis of the changes in the reaction free energy due to surface defects, the vacancy formation energy associated with the Bi vacancies located in the top, middle, and bottom layers (**Figure** S26a) was calculated; the surface model with vacancies in the top layer was used, which resulted in maximum stability. A vacuum space of ~15 Å along the z-direction was included in all surface models to avoid artificial interactions between the periodic replicas. During optimization of the surface models, the bottom three layers were fixed, whereas the top three layers and adsorbates were allowed to relax.

As in our previous DFT studies,[6-8] the free energy of each reaction step was computed at 298.15 K by considering the zero-point energy, entropy, and enthalpy. Additionally, the solvation effect was incorporated using the implicit solvation model available in VASPsol.[9,10] As shown in **Figure** S26b–d, the possible adsorption sites on each surface model were extensively examined for OCHO

adsorption. However, only the most stable OCHO-adsorption configuration was considered in this study. The chemical bonding properties were analyzed by using the projected crystal orbital Hamilton population (COHP) analysis method available in the LOBSTER software package.[11]

Supplementary figures

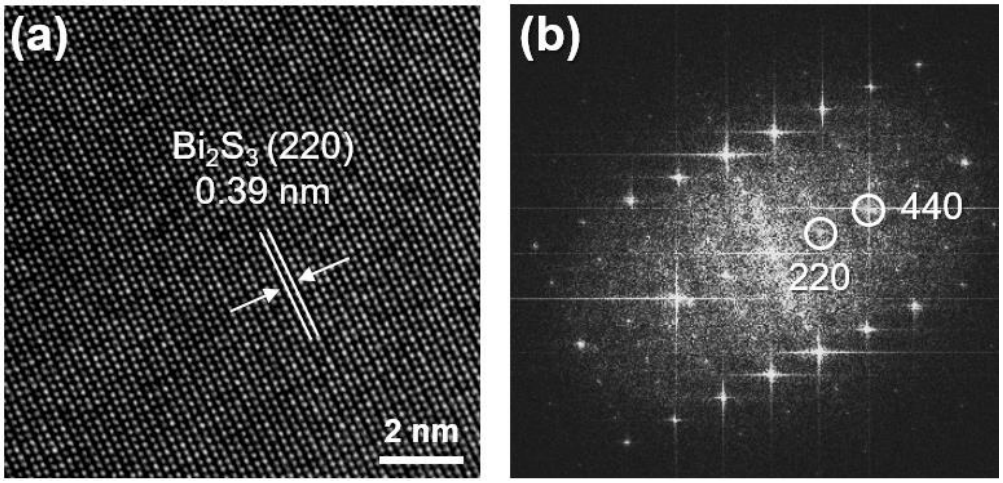

Figure S1. (a) HRTEM image and (b) FFT of Bi_2S_3.

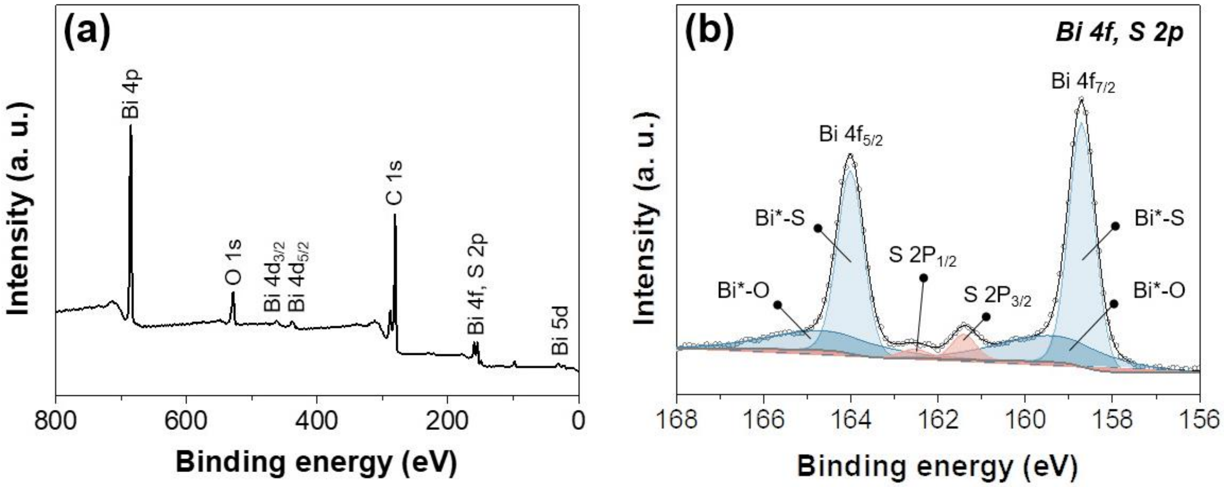

Figure S2. XPS spectra of Bi_2S_3 nanorods: (a) survey of the sample, (b) Bi 4f and S 2p.

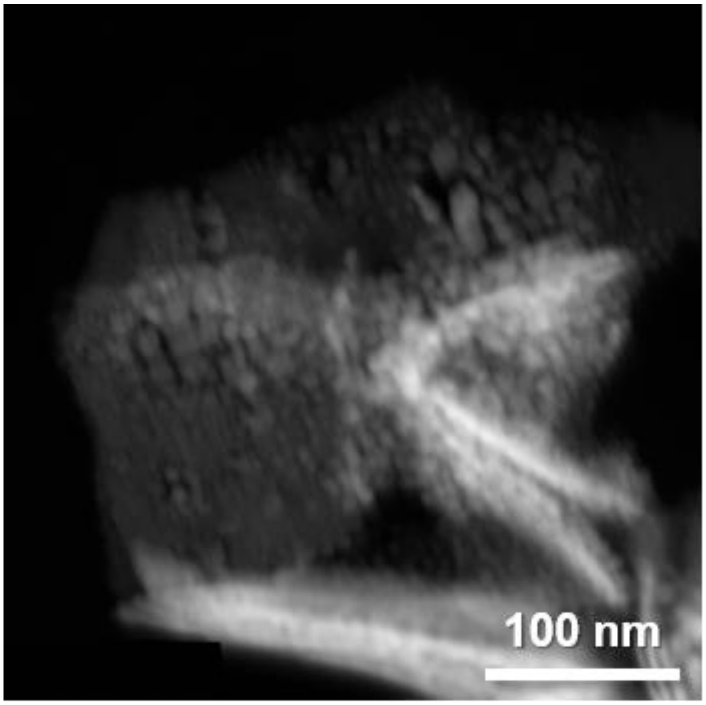

Figure S3. Dark-field HRTEM images of S-Bi nanoplates.

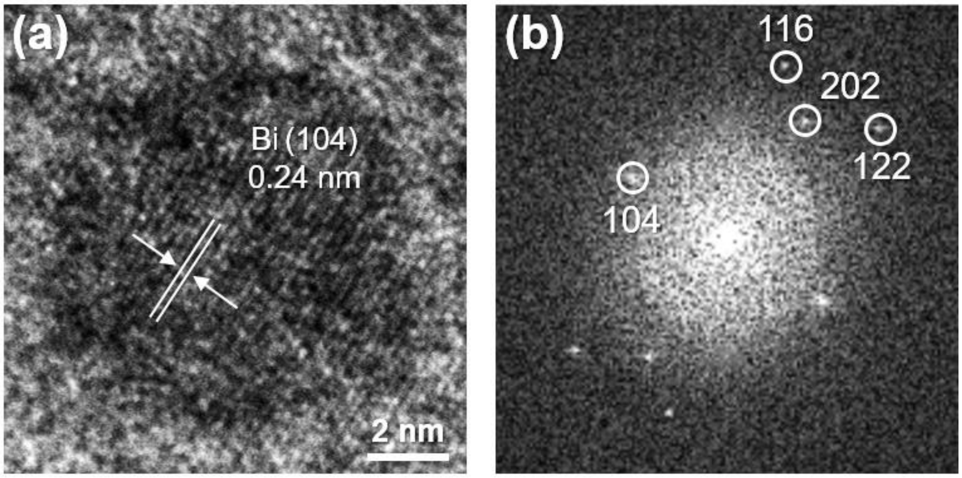

Figure S4. (a) HRTEM image and (b) FFT of S-Bi.

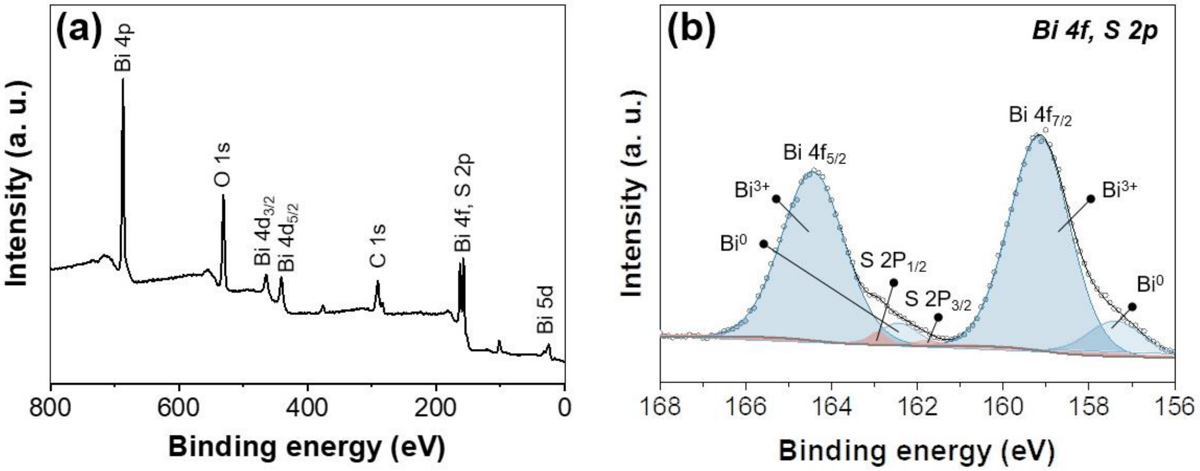

Figure S5. XPS spectra of S-Bi/CP: (a) survey of the sample, (b) Bi 4f and S 2p.

(a)

Element	Before [Norm. wt.%]	After [Norm. wt.%]
Bi	97.56	88.70
S	0.32	0.41
O	2.12	10.89

(b)

Element	PPM	wt.%
Bi	273.16	99.34
S	1.83	0.66

Figure S6. (a) SEM-EDS and (b) ICP-OES analysis of S-Bi catalyst after E-CO_2RR.

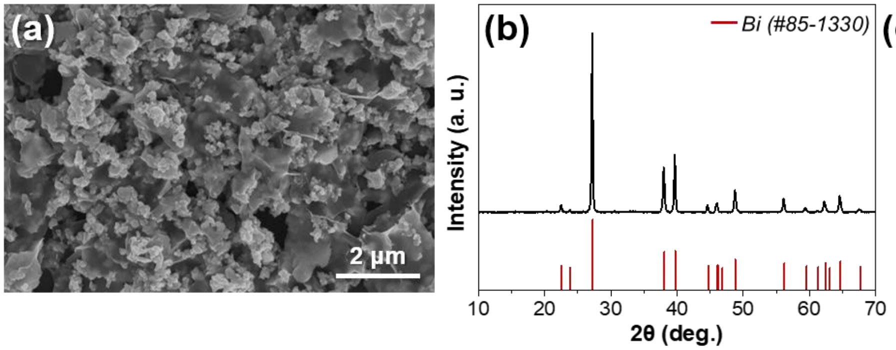

(c)

Element	Wt.%
Bi	86.78
S	0.00
O	13.22

Figure S7. (a) SEM image and (b) XRD pattern of bare Bi nanoparticles. (c) SEM-EDS of bare Bi catalyst after electrochemical reduction.

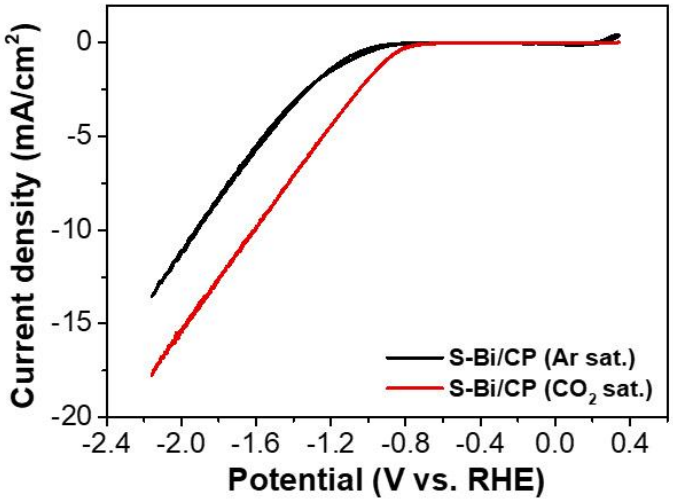

Figure S8. CVs of S-Bi/CP in Ar- (black) and CO_2-saturated (red) 0.1 M $KHCO_3$ electrolyte with a scan rate of 10 mV/s.

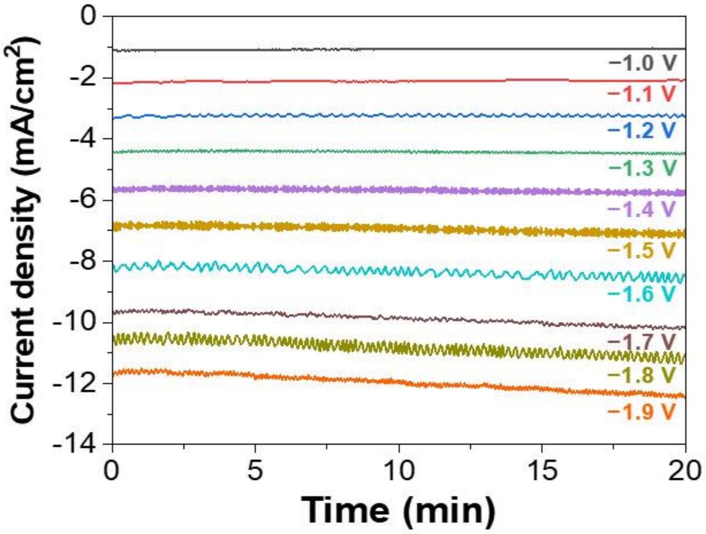

Figure S9. Chronoamperometry curve of S-Bi/CP depending on applied potentials in CO_2-saturated 0.1 M $KHCO_3$ electrolyte.

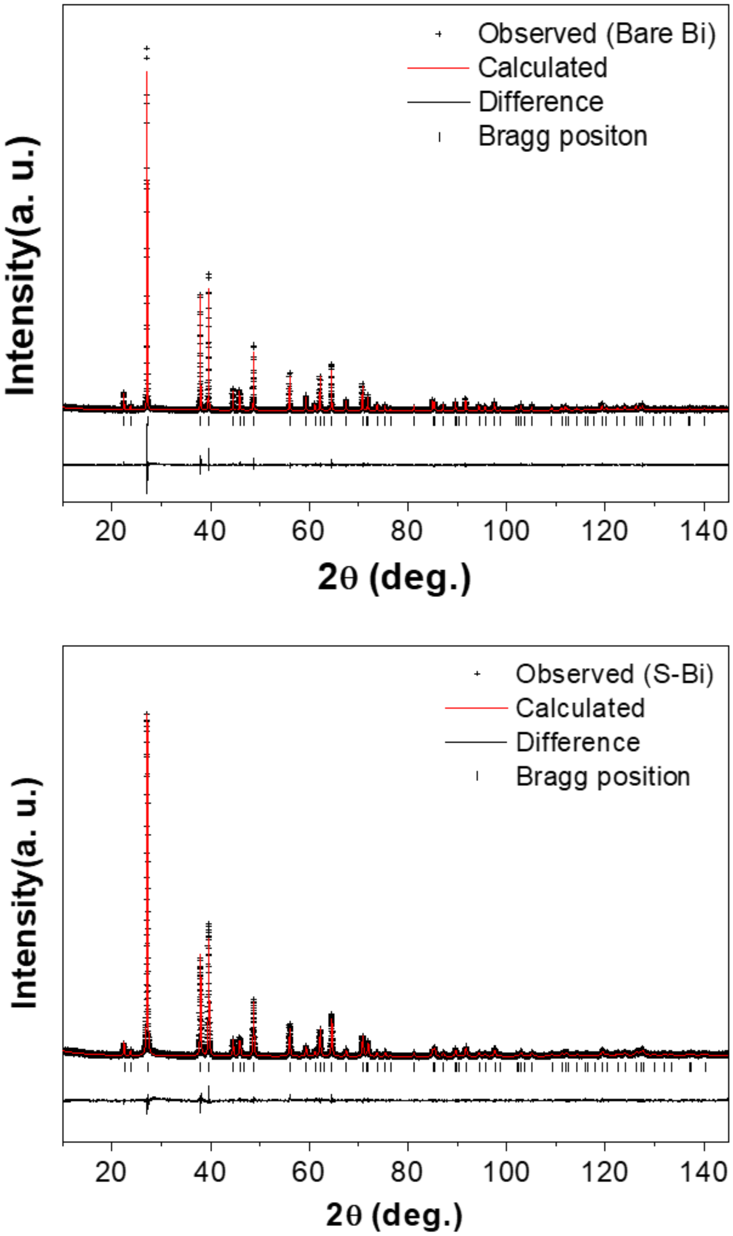

Figure S10. The refined diffraction patterns of the Bi samples (top: Bare Bi and bottom: S-Bi) using X-ray powder diffraction data: observed pattern (dotted curve) and calculated pattern (continuous curve). The diagram underneath shows the difference between them. The ticks show the position of the Bragg reflections of Bi for the Bi samples, respectively.

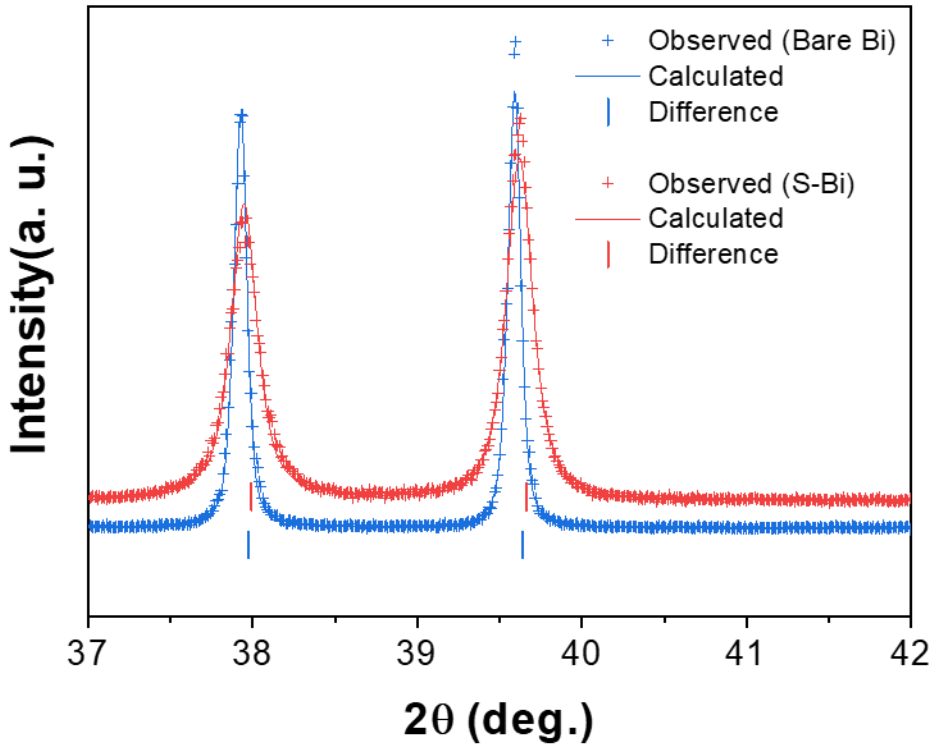

Figure S11. A part of the refined diffraction patterns for the Bi samples (Blue: Bare-Bi and Red: S-Bi) using X-ray powder diffraction data: observed pattern (dotted curve) and calculated pattern (continuous curve). The ticks show the position of the Bragg reflections of Bi for the Bi samples, respectively.

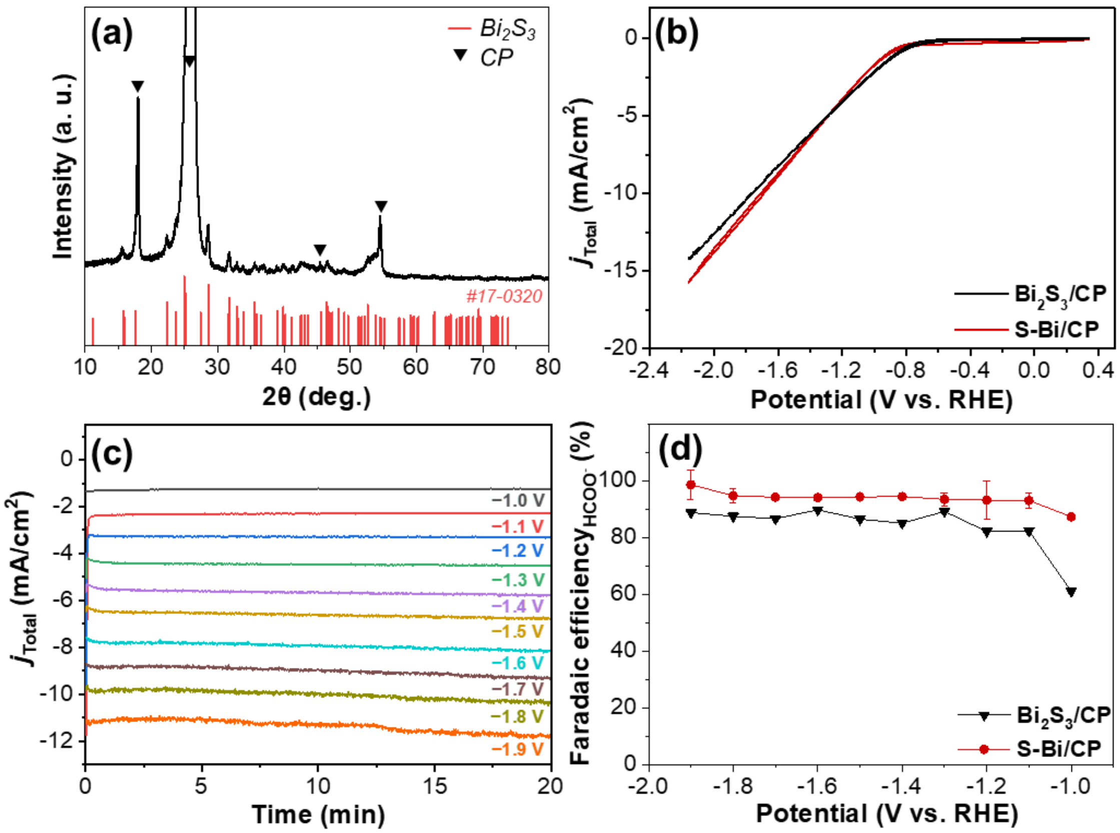

(e)

Element	Applied potential of -1.0 V	Applied potential of -1.9 V
**Bi**	67.06	62.03
**S**	1.19	0.93
**O**	31.75	37.04

Figure S12. (a) XRD pattern of Bi_2S_3/CP. E-CO_2RR efficiency of Bi_2S_3/CP 0.1 M $KHCO_3$. (b) CVs of Bi_2S_3/CP (black) and S-Bi/CP (red). (c) Chronoamperometry curve of Bi_2S_3/CP depending on applied potentials in CO_2 saturated 0.1 M $KHCO_3$. (d) Faradaic efficiency of $HCOO^-$ using Bi_2S_3/CP (black) and S-Bi/CP (red) (blue: $HCOO^-$, red: CO, green: H_2) depending on applied potentials. (e) SEM-EDS analysis of Bi_2S_3/CP electrode before and after E-CO_2RR.

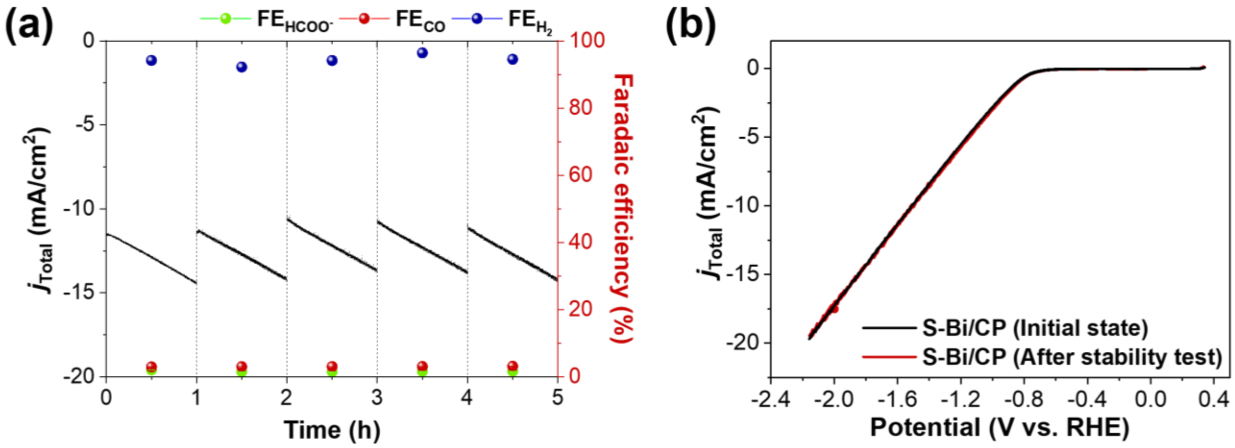

Figure S13. (a) Chronoamperimetric data and Faradaic efficiency of S-Bi/CP at an applied potential of -1.9 V vs. RHE over 5 h. (b) CVs of S-Bi/CP before (black) and after (red) CO_2RR in 0.1 M $KHCO_3$ electrolyte.

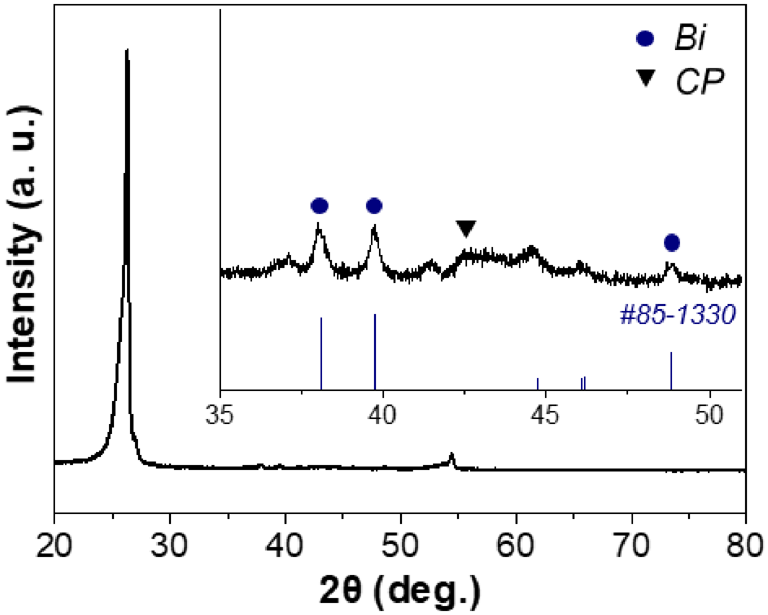

Figure S14. XRD pattern of S-Bi/CP after E-CO_2RR.

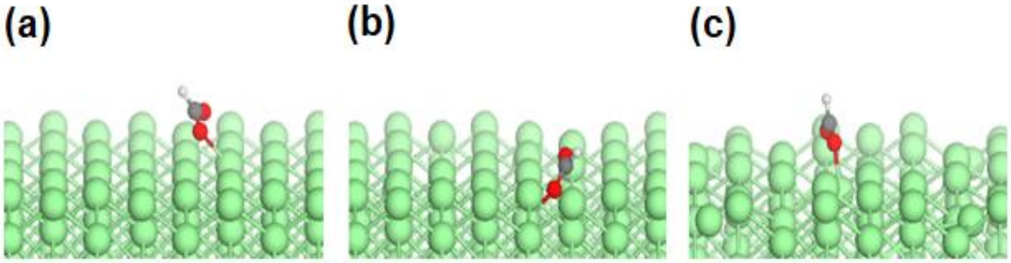

Figure S15. Three-dimensional side views of DFT-optimized OCHO adsorption structures on (a) pristine Bi surface, and defective Bi surfaces with (b) mono-vacancy and (c) di-vacancy.

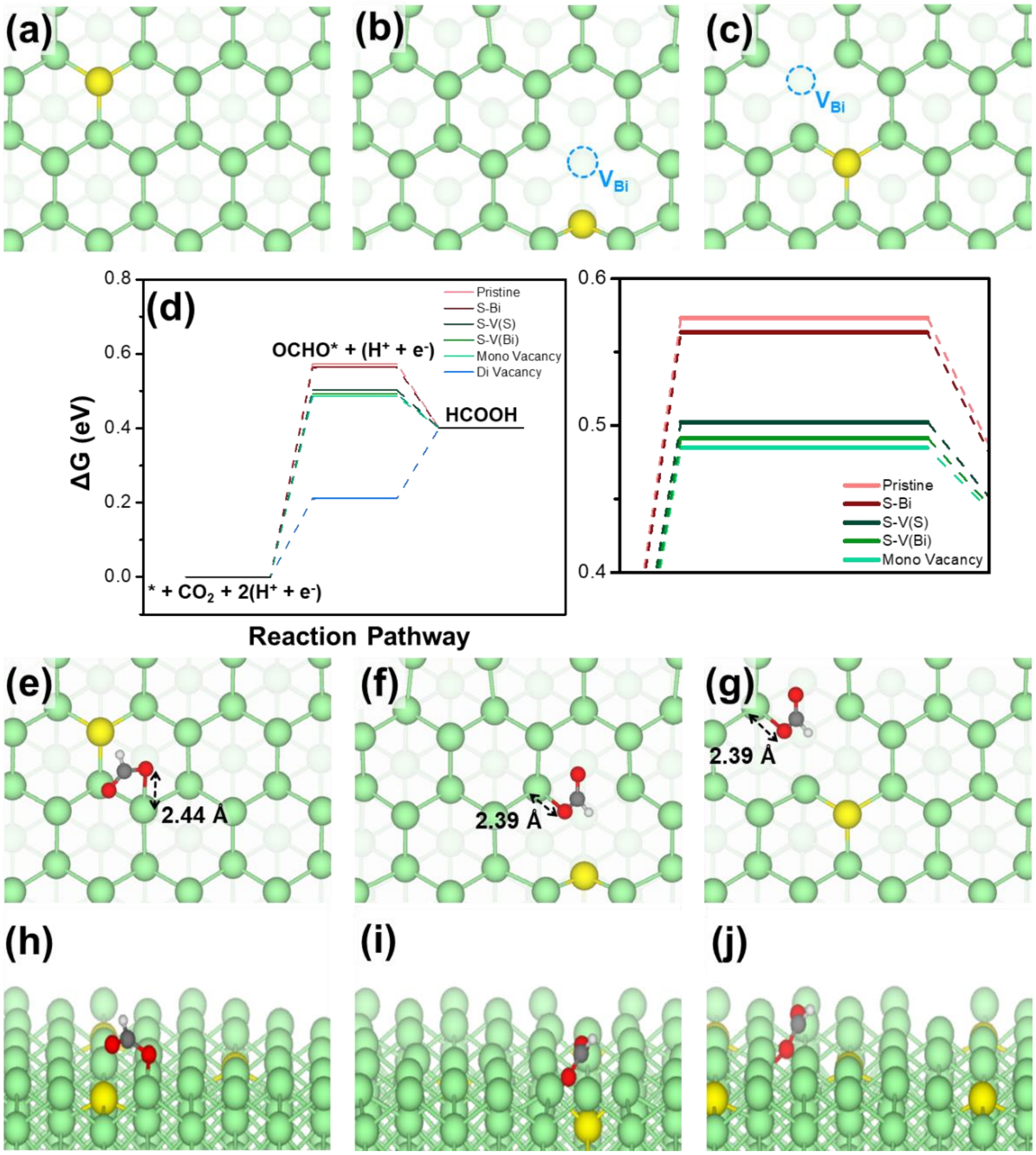

Figure S16. DFT-optimized structures of (a) S-doped pristine Bi surface (S-Bi), (b) S-doped defective Bi surface with the S atom bonded two Bi atoms (S-V(S)), and (c) S-doped defective Bi surface with the S atom bonded to three Bi atoms (S-V(Bi)). (d) Free-energy diagrams for CO_2RR to generate formate on both pristine and defective Bi surfaces, with and without S doping. DFT-optimized OCHO adsorption structures on (e) S-Bi, (f) S-V(S) and (g) S-V(Bi), along with three-dimensional side views for these structures shown in (h) S-Bi, (i) S-V(S) and (j) S-V(Bi). The green, red, gray, white and yellow balls represent Bi, O, C, H, and S respectively.

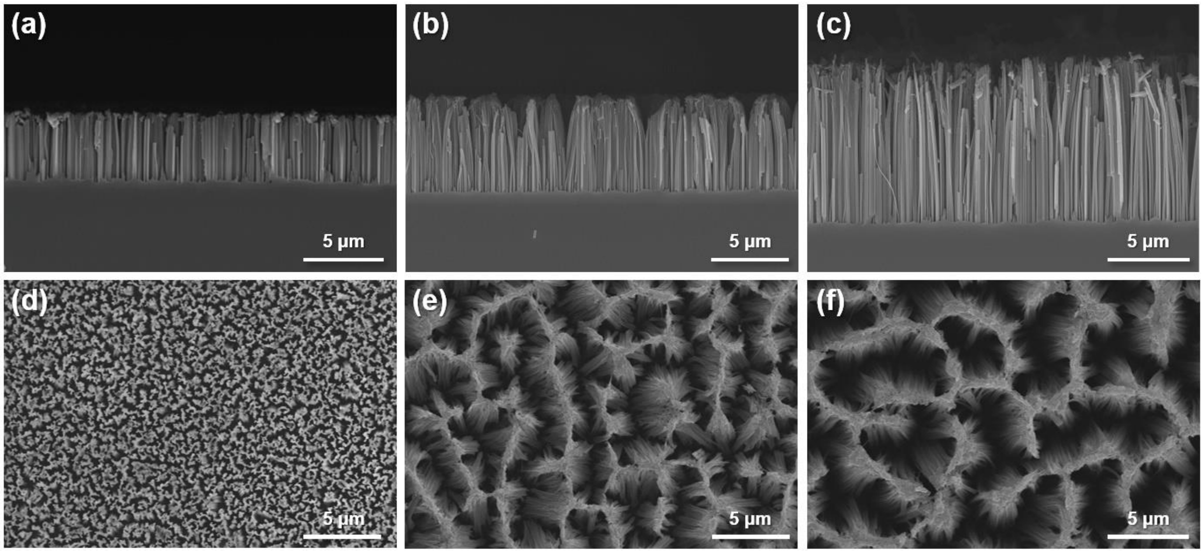

Figure S17. Cross sectional and top view SEM images of SiNWs of (a), (d) 3 µm, (b), (e) 6 µm, (c), (f) 12 µm.

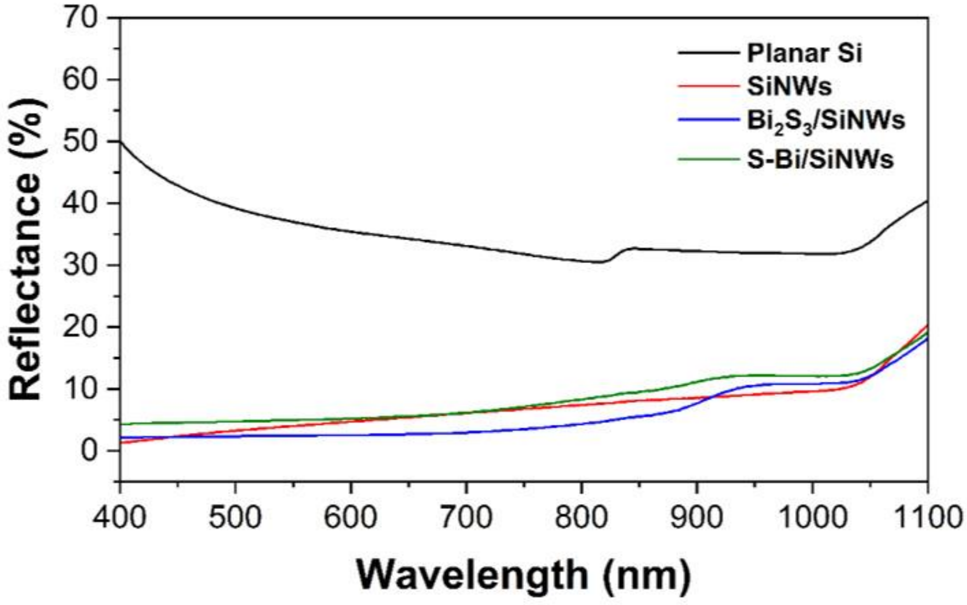

Figure S18. UV-vis reflectance of planar Si, SiNWs, Bi_2S_3/SiNWs and S-Bi/SiNWs.

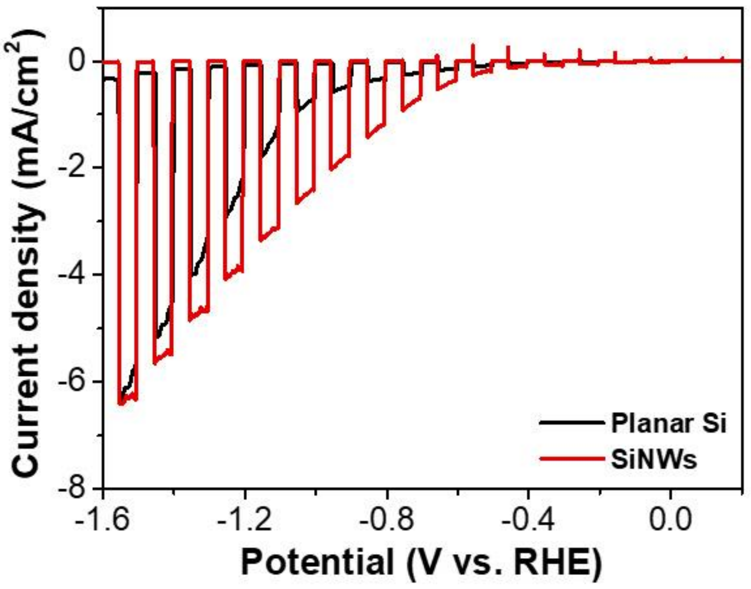

Figure S19. LSVs of planar p-Si (black) and SiNWs (red) in CO_2-saturated 0.1 M $KHCO_3$ electrolyte under UV-vis irradiation with a scan rate of 10 mV/s (light intensity: 100 mW/cm^2).

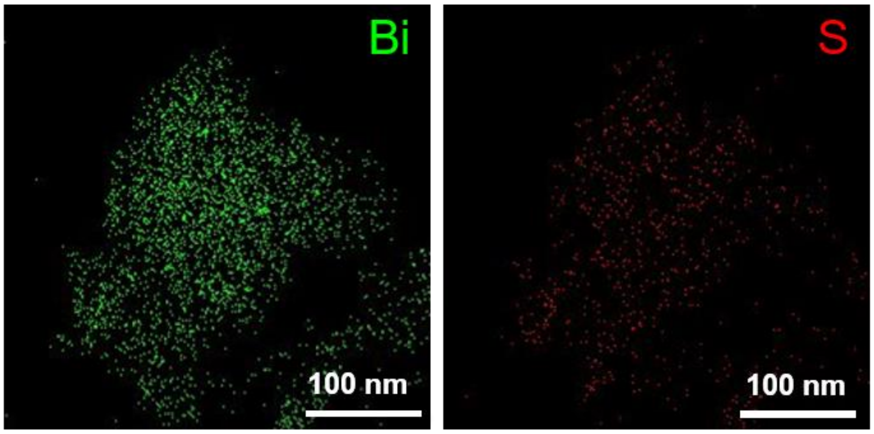

Element	Before [Norm. wt.%]	Error in wt % (Sigma)
**Bi**	94.12	0.11
**S**	0.25	0.06
**O**	5.63	0.09

Figure S20. TEM-EDS analysis data of S-Bi on the SiNWs after photoelectrochemical reduction.

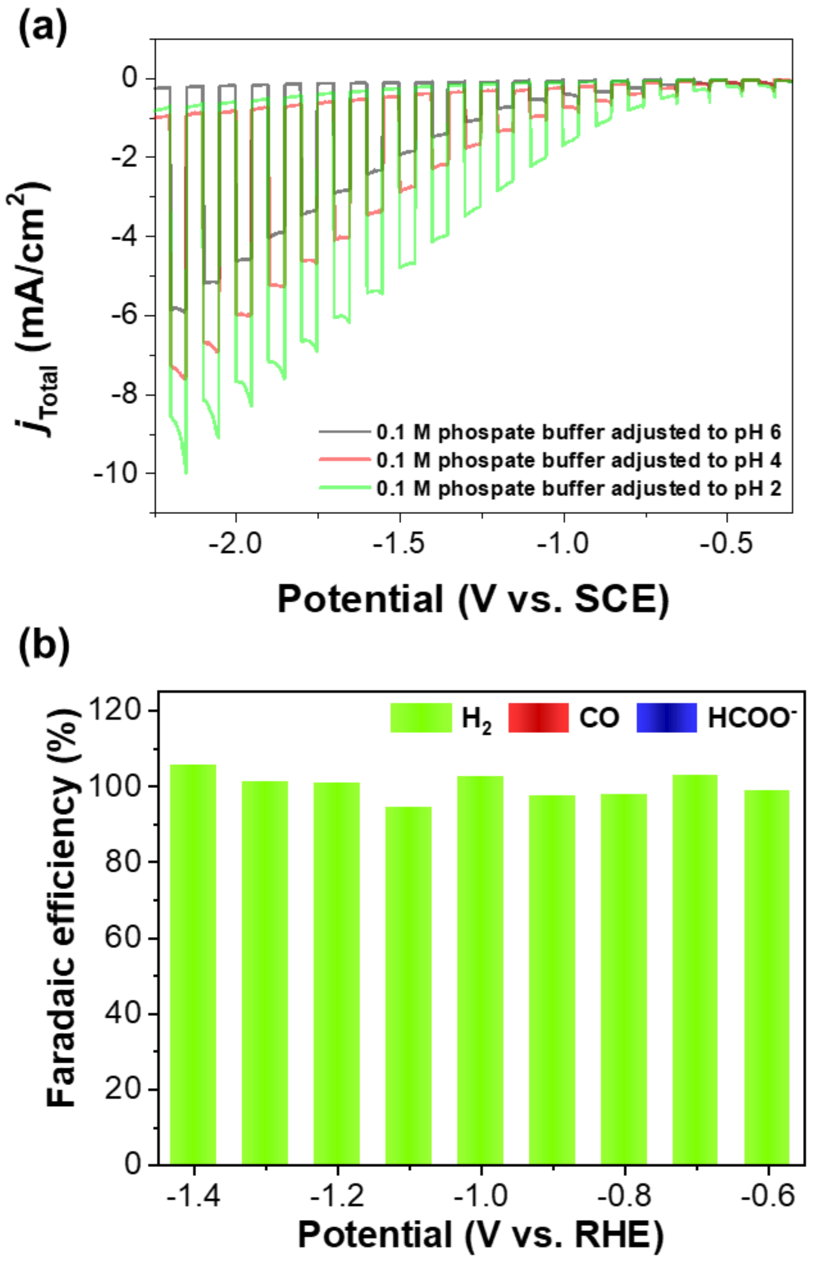

Figure S21. (a) LSVs of SiNWs in 0.1 M phosphate buffer adjusted to pH 6 (black), pH 4 (red), pH 2 (green) under UV-vis irradiation with a scan rate of 10 mV/s (light intensity: 100 mW/cm^2). (b) Faradaic efficiency of SiNWs (blue: $HCOO^-$, red: CO, green: H_2) depending on applied potentials in CO_2-saturated 0.1 M $KHCO_3$ electrolyte.

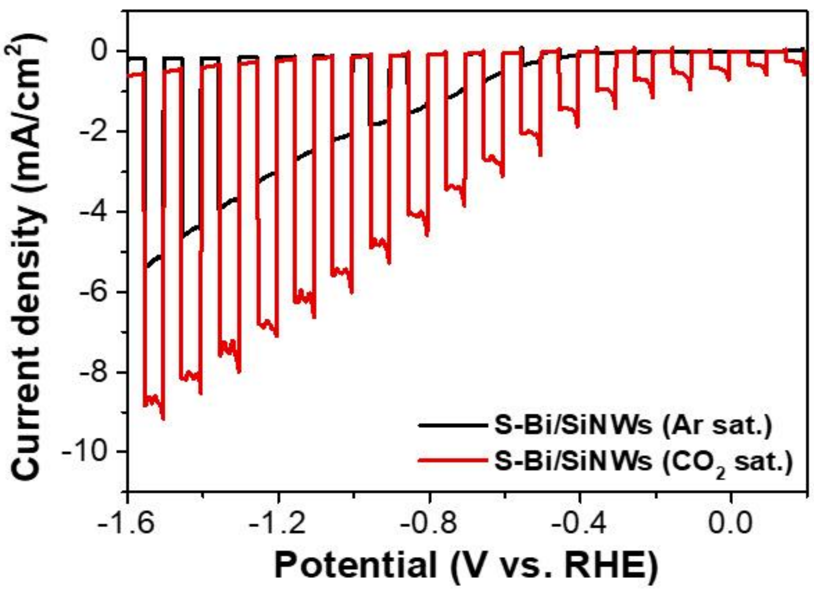

Figure S22. LSVs of S-Bi/SiNWs in Ar- (black) and CO_2-saturated (red) 0.1 M $KHCO_3$ electrolyte under UV-vis irradiation with a scan rate of 10 mV/s (light intensity: 100 mW/cm^2).

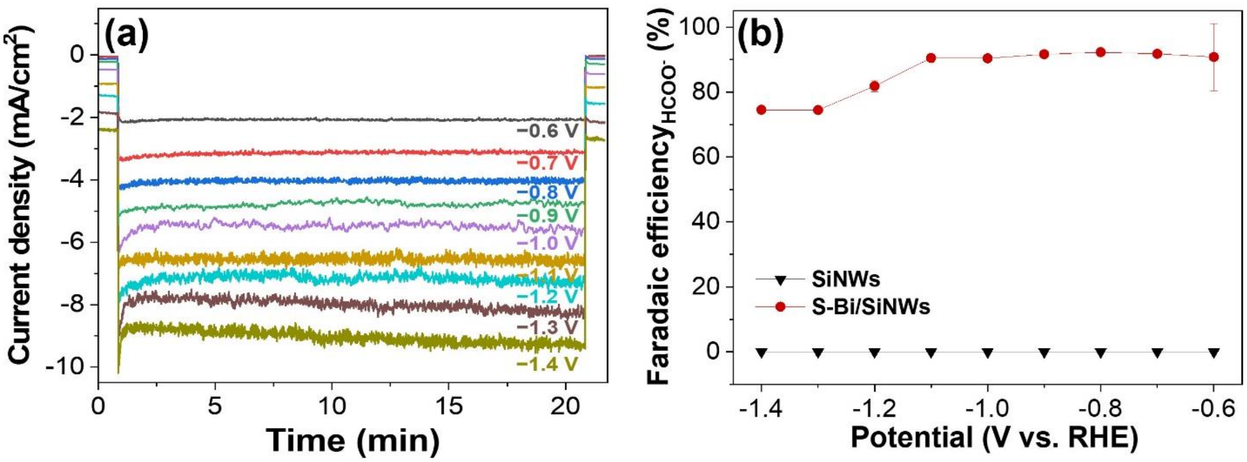

Figure S23. (a) Chronoamperometry curve of S-Bi/SiNWs depending on applied potentials. (b) $HCOO^-$ Faradaic efficiency of S-Bi/SiNWs (red) and SiNWs (black) depending on applied potentials in CO_2-saturated 0.1 M $KHCO_3$ electrolyte.

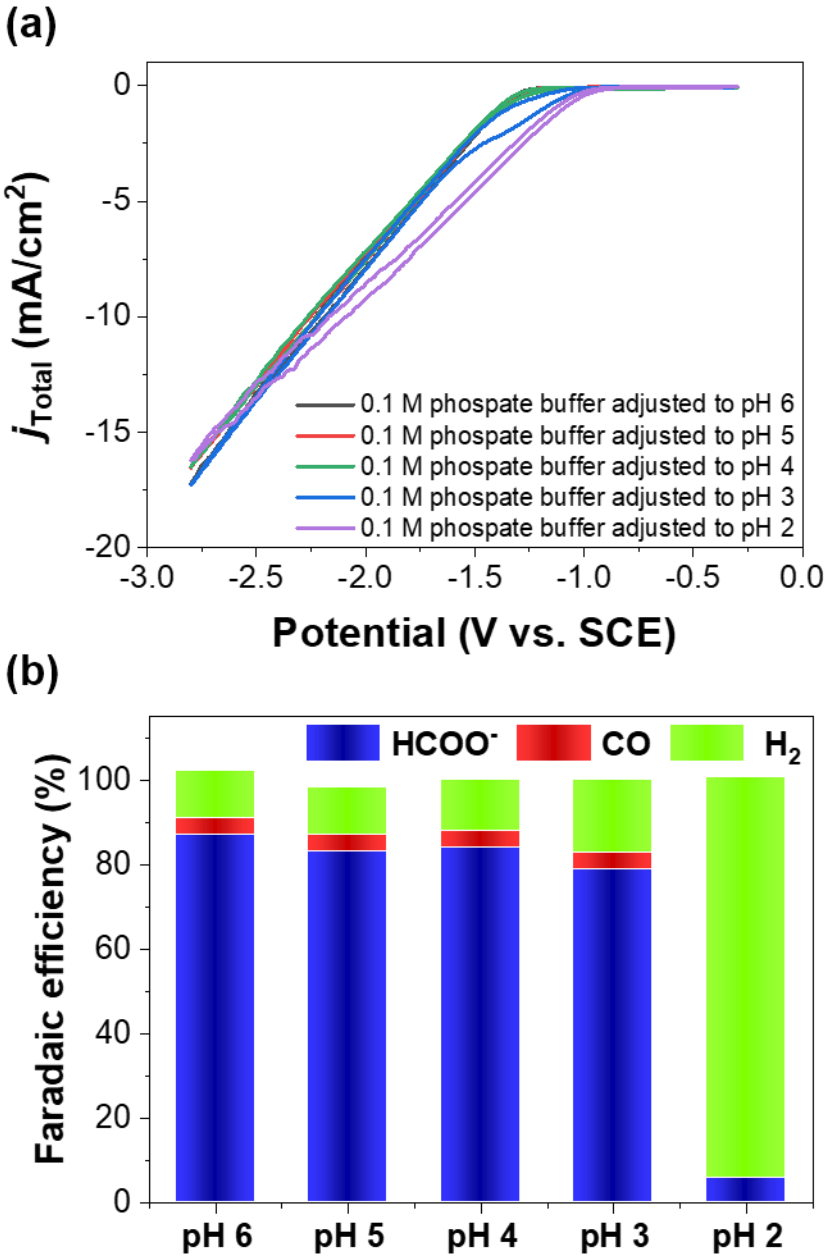

Figure S24. (a) LSVs of S-Bi/CP in 0.1 M phosphate buffer adjusted to pH 6 (black), pH 5 (red), pH 4 (green), pH 3 (blue), and pH 2 (purple). (b) $HCOO^-$ Faradaic efficiency of S-Bi/CP in CO_2-saturated 0.1 M phosphate buffer under varying pH conditions.

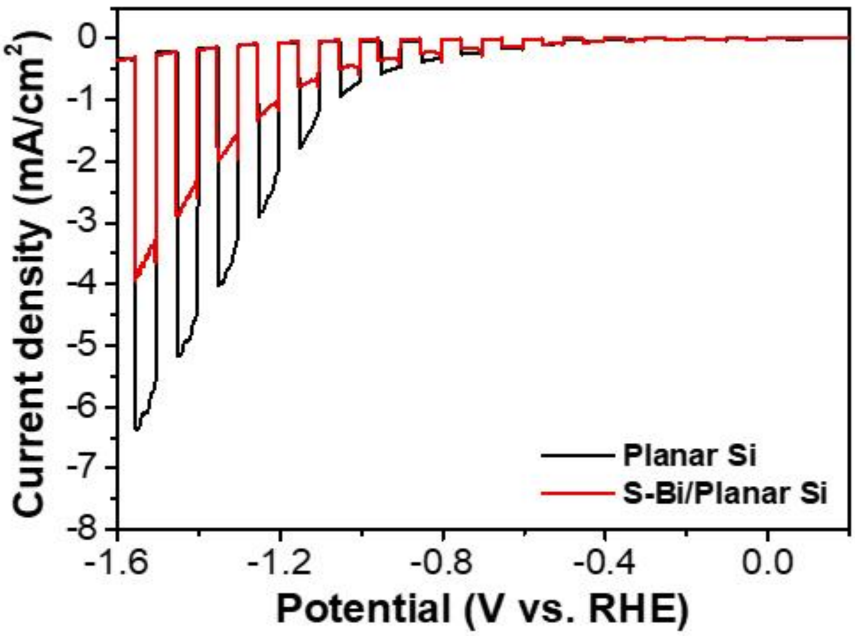

Figure S25. LSVs of planar Si (black) and S-Bi/ planar Si (red) in CO_2-saturated 0.1 M $KHCO_3$ electrolyte under UV-vis irradiation with a scan rate of 10 mV/s (light intensity: 100 mW/cm^2).

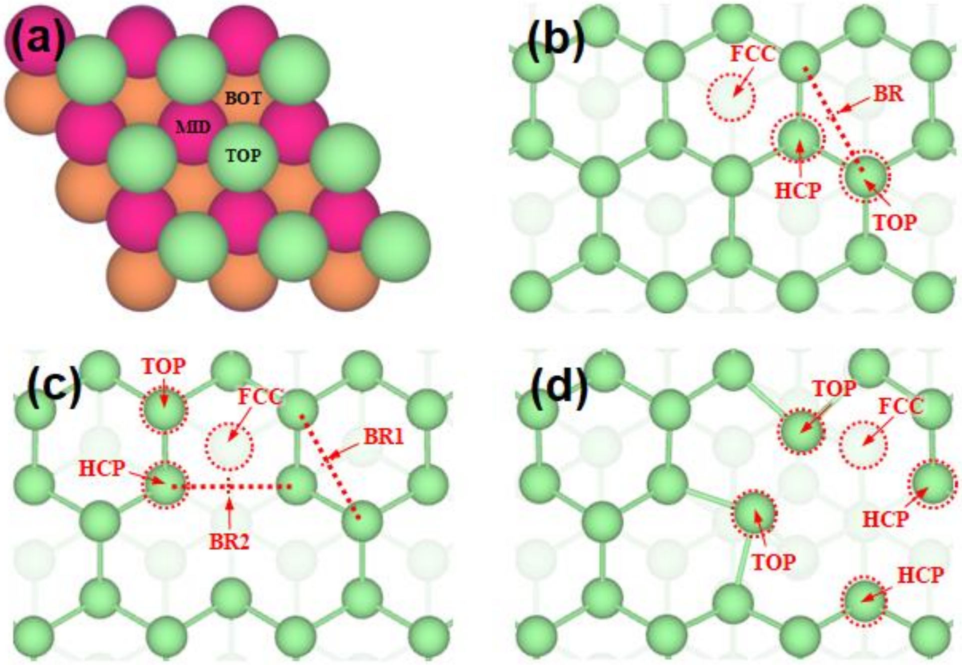

Figure S26. Illustration of (a) Bi vacancy sites on the pristine Bi (001) surface, utilized in the modelling of the defective Bi surfaces. Adsorption sites of OCHO on the (b) pristine Bi (001) surface, (c) defective Bi (001) surface with mono-vacancy, and (d) defective Bi (001) surface with di-vacancy.

Table S1. Comparative analysis of Bi-based materials for CO_2RR activity

Materials	Synthesis method	Loading mass	Precursors	Electrolyte	Product	Current density (mA/cm^2)	FE (%)	Ref.
S-doped Bi nanoplates	Electrochemical reduction	0.2 mg/cm^2	Bi_2S_3	0.1 M $KHCO_3$	Formate	12.7	94.4 % at −1.9 V vs. RHE (>93% at −1.1 V~−1.9 V vs. RHE)	**This work**
Bi nanosheets	In situ transformation	0.3 mg/cm^2	BiOCl	0.5 M $KHCO_3$	Formate	10.5	92 % at −0.9 V vs. RHE	[12]
Bi nanosheets	Hydrothermal method	0.3 mg/cm^2	$Bi(NO_3)_3 \cdot 5H_2O$	0.1 M $KHCO_3$	Formate	6.2	85 % at −0.85 V vs. RHE	[13]
Bi nanowires	Hydrothermal method	0.3 mg/cm^2	$NaBiO_3 \cdot 2H_2O$	0.1 M $KHCO_3$	Formate	-	74 % at −0.85 V vs. RHE	[13]
Bi nanospheres	Hydrothermal method	0.3 mg/cm^2	$NaBiO_3 \cdot 2H_2O$	0.1 M $KHCO_3$	Formate	-	58 % at −0.85 V vs. RHE	[13]
Bi nanosheets /Cu foam	In situ transformation	-	$Bi_2O_2CO_3$ /Cu foam	0.1 M $KHCO_3$	Formate	10.3	92.3% at −1.1 V vs. RHE	[14]
Bi_2S_3-Bi_2O_3 nanosheets	Wet chemical method	1.0 mg/cm^2	$Bi(NO_3)_3 \cdot 5H_2O$	0.1 M $KHCO_3$	Formate	17.1	93.8% at −1.1 V vs. RHE	[15]
Bi_2S_3-Bi_2O_3 nanosheets	Partial precipitation conversion [illegible]	0.4 mg/cm^2	$Bi(NO_3)_3 \cdot 5H_2O$	0.1 M $KHCO_3$	Formate	6.14	90.1% at −0.9 V vs. RHE	[16]
CeO_2/Bi_2S_3 nanorods	Hydrothermal method	-	$Bi(NO_3)_3 \cdot 5H_2O$	0.5 M $KHCO_3$	Formate	≈ 13.3	95.6% at −0.93 V vs. RHE	[17]
Bi_2S_3 nanosheets	Chemical vapor sulfurization	1.0 mg/cm^2	Bi_2O_3	0.5 M $KHCO_3$	Formate	-	98.6% at −0.86 V vs. RHE	[18]
Bi nanosheets	Electrochemical reduction	0.25 g/cm^2	Bi_2S_3	0.5 M $NaHCO_3$	Formate	3.95	90% at −0.9 V vs. RHE	[19]
Bi nanosheets	Liquid phase exfoliation	2.0 mg/cm^2	Bulk Bi	0.1 M $KHCO_3$	Formate	16.5	86% at −1.1 V vs. RHE	[20]
Bi nanosheets	Electrodeposition	3.61 mg/cm^2	$Bi(NO_3)_3 \cdot 5H_2O$	0.1 M $KHCO_3$	Formate	3.5	95.8% at −1.5 V vs. RHE	[21]
Bi nanostructure	Electrochemical reduction	0.1 mg/cm^2	Bi_2S_3	0.5 M $NaHCO_3$	Formate	5	84% at −0.75 V vs. RHE	[22]
BiOCl nanosheets	Hydrothermal method	1.0 mg/cm^2	$Bi(NO_3)_3 \cdot 5H_2O$	0.5 M $KHCO_3$	Formate	11.5	92% at −0.89 V vs. RHE	[23]
Bi nanosheets	Electrochemical transformation	1.0 mg/cm^2	BiOI	0.5 M $KHCO_3$	Formate	17	95.9% at −0.77 V vs. RHE	[24]

Table S2. Refined structural parameters of the Bi samples (bare Bi and S-Bi) using X-ray powder diffraction data at 297 K. The symbols g and B_{eq} represent the occupation factor and the isotropic thermal parameter, respectively. The numbers in parentheses are the estimated standard deviations of the last significant figure.

1) Bare-Bi particles

R_{wp} = 15.64 %, R_p = 11.93 %, and GOF (goodness of fit) = 1.39

Bare-Bi						
Atom	site	x	y	z	g	B_{eq}/Å^2
Bi	6c	0.0	0.0	0.2340(1)	1.0	0.97(2)
Space group: R -3 m (No. 166) and Z = 6						
a (= b) = 4.5439(1) Å and c = 11.8564(4) Å, α (= β) = 90°, β = 120°						

2) S-Bi particles

R_{wp} = 11.79 %, R_p = 8.76 %, and GOF (goodness of fit) = 1.37

S-Bi						
Atom	site	x	y	z	g	B_{eq}/Å^2
Bi	6c	0.0	0.0	0.2340(1)	0.981(2)	1.07(2)
Space group: R -3 m (No. 166) and Z = 6						
a (= b) = 4.5412(2) Å and c = 11.8529(5) Å, α (= β) = 90°, β = 120°						

REFERENCES

(1) G. Kresse, J. Furthmüller, *Phys. Rev. B Condens. Matter* **1996**, 54, 11169.

(2) P. E. Blöchl, *Phys. Rev. B* **1994,** 50, 17953.

(3) J. P. Perdew, K. Burke, M. Ernzerhof, *Phys. Rev. Lett.* **1996**, 77, 3865.

(4) S. Grimme, J. Antony, S. Ehrlich, H. Krieg, *J. Chem. Phys.* **2010,** 132, 154104.

(5) P. Hofman, *Prog. Surf. Sci.* **2006**, 81, 191.

(6) R. S. Kanase, G. M. Zewdie, M. Arunachalam, J. Badiger, S. A. Sayed, K. Ahn, J. Ha, U. Sim, H. Shin, S. H. Kang, *J. Energy Chem.* **2024**, 88, 71.

(7) Y. Sun, H. Shin, F. Wang, B. Tian, C.-W. Chiang, S. Liu, X. Li, Y. Wang, L. Tang, W. A. Goddard Ill, M. Ding, *J. Am. Chem. Soc.* **2022**, 144, 33, 15185.

(8) W. Jung, J. Jeong, Y. Chae, W. H. Lee, Y.-J. Ko, K. H. Chae, H.-S. Oh, U. Lee, D. K. Lee, B. K. Min, H. Shin, Y. J. Hwang, D. H. Won, *J. Mater. Chem. A* **2022**, 10, 23760.

(9) K. Mathew, V. S. Chaitanya Kolluru, S. Mula, S. N. Steinmann, R. G. Hennig, *J. Chem. Phys.* **2019**, 151, 234101.

(10) K. Mathew, R. Sundararaman, K. Letchworth-Weaver, T. A. Arias, R. G. Hennig, *J. Chem. Phys.* **2014**, 140, 084106.

(11) S. Maintz, V. L. Deringer, A. L. Tchougréeff, R. Dronskowski, *J. Comput. Chem.* **2016**, 37, 1030.

(12) Y. Guan, M. Liu, X. Rao, Y. Liu, J. Zhang, *J. Mater. Chem. A* **2021**, 9, 13770.

(13) T. Gao, X. Wen, T. Xie, N. Han, K. Sun, L. Han, H. Wang, Y. Zhang, Y. Kuang, X. Sun, *Electrochim. Acta* **2019**, 305, 388.

(14) X. An, S. Li, X. Hao, X. Du, T. Yu, Z. Wang, X. Hao, A. Abudula, G. Guan, *Sustain. Energy Fuels* **2020**, 4, 2831.

(15) P. Sui, C. Xu, M. Zhu, S. Liu, Q. Liu, J. Luo, *Small* **2022**, 18, 2105682.

(16) X. Yang, P. Deng, D. Liu, S. Zhao, D. Li, H. Wu, Y. Ma, B. Y. Xia, M. Li, C. Xiao, S. Ding, *J. Mater. Chem. A* **2020**, 8, 2472.

(17) S. Wu, M. Tian, Y. Hu, N. Zhang, W. Shen, J. Li, L. Guo, P. Da, P. Xi, C. Yan, *Inorg. Chem.* **2023**, 62, 4088.

(18) Y. Li, J. Chen, S. Chen, T. Lu, X. Liao, T. Zhao, F. Cheng, H. Wang, *Appl. Catal. B Environ.* **2024**, 349, 123870.

(19) P. Su, W. Xu, Y. Qiu, T. Zhang, X. Li, H. Zhang, *ChemSusChem* **2018**, 11, 848.

(20) W. Zhang, Y. Hu, L. Ma, G. Zhu, P. Zhao, X. Xue, R. Chen, S. Yang, J. Ma, J. Liu, Z. Jin, *Nano Energy* **2018**, 53, 808.

(21) H. Jiang, L. Wang, Y. Li, B. Gao, Y. Guo, C. Yan, M. Zhuo, H. Wang, S. Zhao, *Appl. Surf. Sci.* **2021**, 541, 148577.

(22) Y. Zhang, F. Li, X. Zhang, T. Williams, C. D. Easton, A. M. Bond, J. Zhang, *J. Mater. Chem. A* **2018**, 6, 4714.

(23) J. Zhu, J. Fan, T. Cheng, M. Cao, Z. Sun, R. Zhou, L. Huang, D. Wang, Y. Li, Y. Wu, *Nano Energy* **2020**, 75, 104939.

(24) D. Wu, J. Liu, Y. Liang, K. Xiang, X. Fu, J. Luo, *ChemSusChem* **2019**, 12, 4700.
